# Integrative Genome-Wide Expression Analysis Bears Evidence of Estrogen Receptor-Independent Transcription in Heregulin-Stimulated MCF-7 Cells

**DOI:** 10.1371/journal.pone.0001803

**Published:** 2008-03-19

**Authors:** Takeshi Nagashima, Takahiro Suzuki, Shinji Kondo, Yoko Kuroki, Kaoru Takahashi, Kaori Ide, Noriko Yumoto, Aki Hasegawa, Tetsuro Toyoda, Toshio Kojima, Akihiko Konagaya, Harukazu Suzuki, Yoshihide Hayashizaki, Yoshiyuki Sakaki, Mariko Hatakeyama

**Affiliations:** 1 Computational and Experimental Systems Biology Group, RIKEN Genomic Sciences Center, RIKEN Yokohama Institute, Yokohama, Japan; 2 Genome Exploration Research Group, RIKEN Genomic Sciences Center, RIKEN Yokohama Institute, Yokohama, Japan; 3 Advanced Genome Information Technology Research Group, RIKEN Genomic Sciences Center, RIKEN Yokohama Institute, Yokohama, Japan; 4 Division of Genomic Information Resources, Supramolecular Biology, International Graduate School of Arts and Sciences, Yokohama City University, Yokohama, Japan; 5 Genome Science Laboratory, Discovery and Research Institute, RIKEN Wako Main Campus, Saitama, Japan; 6 Functional RNA Research Program, RIKEN Frontier Research System, Saitama, Japan; University of Michigan, United States of America

## Abstract

Heregulin ß-1 (HRG) is an extracellular ligand that activates mitogen-activated protein kinase (MAPK) and phosphatidylinositol-3-OH kinase (PI3K)/Akt signaling pathways through ErbB receptors. MAPK and Akt have been shown to phosphorylate the estrogen receptor (ER) at Ser-118 and Ser-167, respectively, thereby mimicking the effects of estrogenic activity such as estrogen responsive element (ERE)-dependent transcription. In the current study, integrative analysis was performed using two tiling array platforms, comprising histone H3 lysine 9 (H3K9) acetylation and RNA mapping, together with array comparative genomic hybridization (CGH) analysis in an effort to identify HRG-regulated genes in ER-positive MCF-7 breast cancer cells. Through application of various threshold settings, 333 (326 up-regulated and 7 down-regulated) HRG-regulated genes were detected. Prediction of upstream transcription factors (TFs) and pathway analysis indicated that 21% of HRG-induced gene regulation may be controlled by the MAPK cascade, while only 0.6% of the gene expression is controlled by ERE. A comparison with previously reported estrogen (E2)-regulated gene expression data revealed that only 12 common genes were identified between the 333 HRG-regulated (3.6%) and 239 E2-regulated (5.0%) gene groups. However, with respect to enriched upstream TFs, 4 common TFs were identified in the 14 HRG-regulated (28.6%) and 13 E2-regulated (30.8%) gene groups. These results indicated that while E2 and HRG may induce common TFs, the regulatory mechanisms that govern HRG- and E2-induced gene expression differ.

## Introduction

Heregulin ß-1 (HRG) is an extracellular ligand that binds to and activates ErbB3 and ErbB4 receptors in a process associated with the control of cell proliferation and differentiation [1]–[3].

Our previous study using MCF-7 breast cancer cells showed that HRG rapidly up-regulated hundreds of transcripts in a ligand dose-dependent manner, as determined by employing expression arrays using the Affymetrix U133A 2.0 chip that mounts protein-coding and non-protein coding regions of ca. 18,400 transcripts [4]. HRG-stimulated ErbB receptor is known to activate mitogen-activated protein kinase (MAPK) and phosphatidylinositol-3-OH kinase (PI3K)/Akt signaling pathways [5]. Consequently, the transcriptional regulation induced by HRG was thought to be controlled by activation of the aforementioned signaling pathways and associated transcription factors (TFs). On the other hand, MAPK and Akt are also known to phosphorylate the estrogen receptor (ER) at Ser-118 and Ser-167, respectively, thereby mimicking the effects of estrogenic activity in ER-positive cells in the absence of estrogen (E2) [6]–[8]. Activated ER is believed to bind directly to the estrogen responsive element (ERE) DNA sequence and subsequently induce ERE-dependent transcription [9]. Given that MCF-7 is an ER-positive breast cancer cell line, HRG-induced transcription may involve both ERE- and non-ERE-dependent regulation.

In our earlier study using the Affymetrix U133A 2.0 chip, however, we found that only 6 transcripts among the 241 HRG-regulated transcripts (2.49%) were derived from genes that possessed the ERE sequence within a 2 kb upstream region of the transcription start site (TSS) [4]. Notwithstanding the fact that HRG-stimulated MCF-7 cells showed ERE-mediated suppression of histone acetylation and gene expression [10], the population of ERE-regulated transcripts in our analysis seemed to be relatively low. This suggested the possibility that use of the expression array platform may have failed to identify the complete range of transcripts associated with HRG-induced gene regulation. In the current study, integrative analysis of HRG-induced gene expression in MCF-7 cells was performed using two tiling array platforms comprising histone H3 lysine 9 (H3K9) acetylation (ChIP-on-chip) and RNA mapping. Array comparative genomic hybridization (CGH) analysis was also employed in an effort to identify gene expression unrelated to HRG treatment.

Integrated analysis of the expression data using various array platforms facilitated the identification of transcripts that were regulated in cells following HRG treatment. The data showed that 326 genes were up-regulated and 7 genes were down-regulated. Prediction of upstream TFs and mapping onto the biological pathways indicated that 21% (69 out of 333) of the genes associated with HRG-induced gene regulation may be controlled by TFs known to be activated by the MAPK cascade, while only 0.6% (2 out of 333) of the gene expression is controlled by ERE.

Comparison with previously reported data pertaining to E2-regulated genes [11], [12] revealed that only 12 common genes were identified between the 333 HRG-regulated (3.6%) and 239 E2-regulated (5.0%) gene groups. However, with respect to upstream TFs that are more likely to be enriched, 4 common TFs were identified in the 14 HRG-induced (28.6%) and 13 E2-induced (30.8%) gene groups. These results indicated that while E2 and HRG may induce common TFs, the regulatory mechanisms that govern HRG- and E2-induced gene expression differ.

## Results

### Stimulation by HRG induces phosphorylation of ER at Ser-118 and Ser-167 in MCF-7 cells

**Figure 1:**
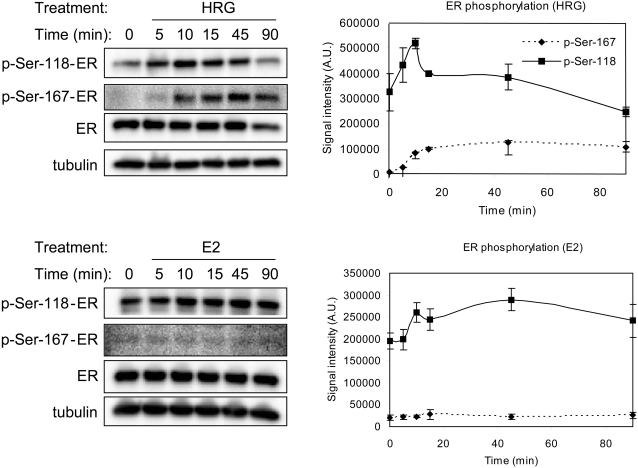
Estrogen receptor phosphorylation induced by HRG and E2. Phosphorylation levels of estrogen receptor alpha at Ser-118 and Ser-167 treated with HRG (10 nM) or E2 (10 nM) were measured at the designated time period. Three-independent western blots were performed for each ligand. Representative figures are shown on the left. Levels of phosphorylation were quantified using a densitometer and are shown graphically on the right.

Modes of ER activation have been categorized largely on the basis of steroid (E2)-dependent and steroid-independent mechanisms. E2 binds directly to ER thereby facilitating ER dimerization and subsequent high affinity binding to ERE (referred to as steroid-dependent ER activation) [13]. Growth hormones such as epidermal growth factor (EGF) and insulin-like growth factor (IGF)-I have been shown to activate ER by phosphorylating ER at Ser-118 through activation of MAPK [6]. On the other hand, Akt and pp90^rsk1^ activated by EGF have been shown to phosphorylate ER at Ser-167 [7], [8], [14]. These steroid-independent activations of ER are also capable of leading to ER- and ERE-mediated transcription events. HRG is a growth hormone ligand that strongly activates MAPK and PI3K/Akt through ErbB receptor activation [1]–[4]. We first assessed an assay to confirm that HRG induces ER phosphorylation at Ser-118 and Ser-167 in MCF-7 cells in our culture condition. The results clearly indicated that HRG treatment immediately induced phosphorylation at both serine residues (Figure 1). These results are consistent with an earlier report showing HRG-dependent ER activation [15]. On the other hand, E2 induced weak phosphorylation at Ser-118 and no phosphorylation at Ser-167. We assumed that ER is strongly activated by HRG under the assay conditions used in this study.

### Comparison of histone acetylation and RNA mapping data with expression array data

HRG-induced gene expression data obtained from various array platforms including the tiling array data was then investigated. These data were submitted to Gene Expression Omnibus (http://www.ncbi.nlm.nih.gov/geo/) and can be accessed through GEO series accession numbers GSE8579 (tiling arrays, histone H3K9 acetylation and RNA mapping) and GSE8471 (expression arrays) and GSE8508 (array CGH)

Although chromatin modification by H3K9 acetylation has been linked to the regulation of gene expression, the precise correlation between the extent of histone acetylation and gene expression remains unclear [16], [17]. On the other hand, although use of RNA mapping on whole-genome tiling arrays seems to represent an unbiased approach [18], this method often yields many false-positive and false-negative values. Therefore, the current study employed an analysis that began with an evaluation of acetylation and RNA map data sets.

In an effort to determine whether the tiling array data accurately reflects the gene expression profile, HRG-mediated up- and down-regulated genes were initially identified from the histone acetylation and RNA map data (see Materials and Methods section). To this end, 27,354 RefSeq transcripts were selected that correspond to 17,843 Entrez Gene IDs (hereafter, the 17,843 genes are referred to as the baseline set). To assess the validity of the gene selection, the derived gene list was compared with genes that were identified in expression arrays as a reference indicator (see Materials and Methods for the selection).

**Figure 2:**
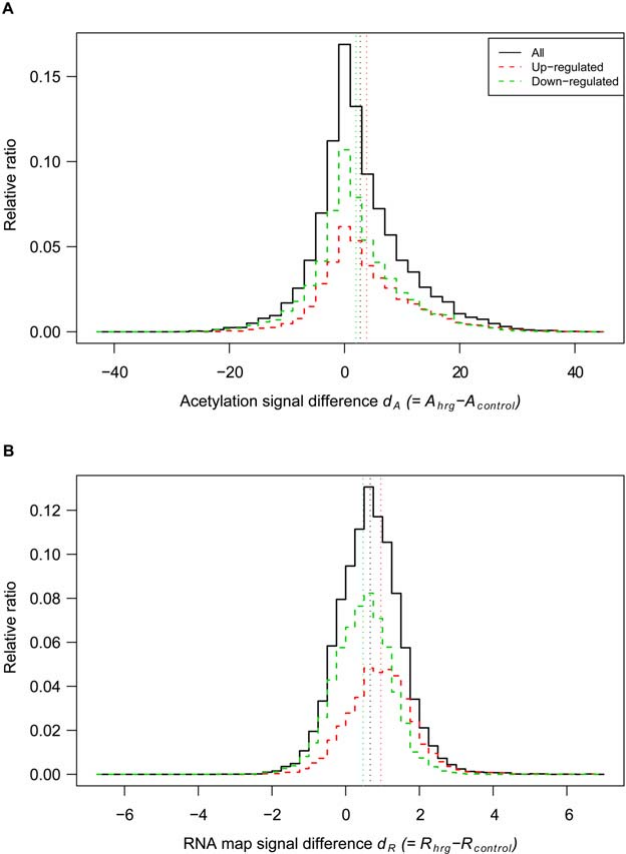
Distribution of signal differences *d_A_* and *d_R_* between HRG-treated and untreated cells. Upper and lower panel shows the distribution of acetylation and RNA map signal differences, *d_A_* ( = *A_hrg_–A_control_*) and *d_R_* ( = *R_hrg_–R_control_*) (A and B), respectively. Larger *d_A_* (or *d_R_*) values represent a higher level of acetylation (or RNA map) in HRG-treated cells compared with untreated cells. Red and green dashed lines show the distribution of these values in the expression array and represent up- and down-regulated genes, respectively. Colored dotted lines depicted along the vertical axis represent average values of each distribution.

HRG-induced up- and down-regulation of genes was assessed by identifying signal differences between HRG-treated and untreated cells. The difference in acetylation signal *d_A_* and that of the RNA map *d_R_* were calculated as follows: *d_A_* = *A_hrg_–A_control_* and *d_R_* = *R_hrg_–R_control_*, where *A_hrg_* and *R_hrg_* represent acetylation and RNA map signals in HRG-treated cells, respectively, and *A_control_* and *R_control_* represent acetylation and RNA map signals in untreated cells, respectively. If *d_A_* or *d_R_* was greater than 0, the gene was regarded as being up-regulated. Figure 2 shows the distribution of these values. The average value of *d_A_* (2.7, black dotted line in Figure 2A) and *d_R_* (0.67, black dotted line in Figure 2B) and the distribution peak were greater than 0, which implies that HRG treatment induced up-regulation of histone acetylation and RNA mapping. If gene expression changes (up- or down-regulation) can be identified by examining acetylation (or RNA map) signals, it is anticipated that this would be reflected in the form of a distinct spread of *d_A_* (or *d_R_*) values relating to up- and down-regulated genes. However, when independently comparing the signal distribution pertaining to the acetylation (or RNA map) data with the expression array results, no clear difference was observed (red and green dashed lines were not distinct in Figure 2). Thus, our analysis revealed that independent tiling array analysis may have failed to identify the complete range of transcripts associated with HRG-induced gene regulation.

In an effort to confirm this possibility, the overlapping rate of genes selected from each tiling array was then compared with the gene list derived from the expression array analysis. For the selection of up-regulated genes from the histone acetylation data, the criterion *A_hrg_*−*A_control_*≥*α* was adopted. If a gene exceeded threshold *α*, such a gene was regarded as being up-regulated. In the same way, *R_hrg_*−*R_control_*≥*β* was used for the RNA map data. Table 1 shows the relative ratio of up-regulated genes that were found in each tiling array data and expression array-based gene list under various thresholds of *α* and *β*. In general, increased thresholds resulted in higher overlapping rates. However, even with such a tight threshold setting, more than 50% of the genes selected from the histone acetylation data were not covered in the gene list obtained from the expression array data. The RNA map data show a higher overlapping rate when the number of genes obtained is quite small (*β* = 4 gives only 29 overlapping genes).

**Table 1:** Comparison of up-regulated genes obtained from each tiling array data with the expression array results.

A Common genes as determined from the acetylation data at various thresholds (*α*)		
*α*	% common genes	No. common genes
0	28.64	3028
2	34.96	2380
4	39.28	1869
6	42.47	1471
8	44.10	1155
10	45.09	881

B Common genes as determined from the RNA map data at various thresholds (*β*)		
*β*	% common genes	No. common genes
0	30.39	3905
1	45.44	2206
2	61.10	476
3	76.67	92
4	90.63	29

**Figure 3:**
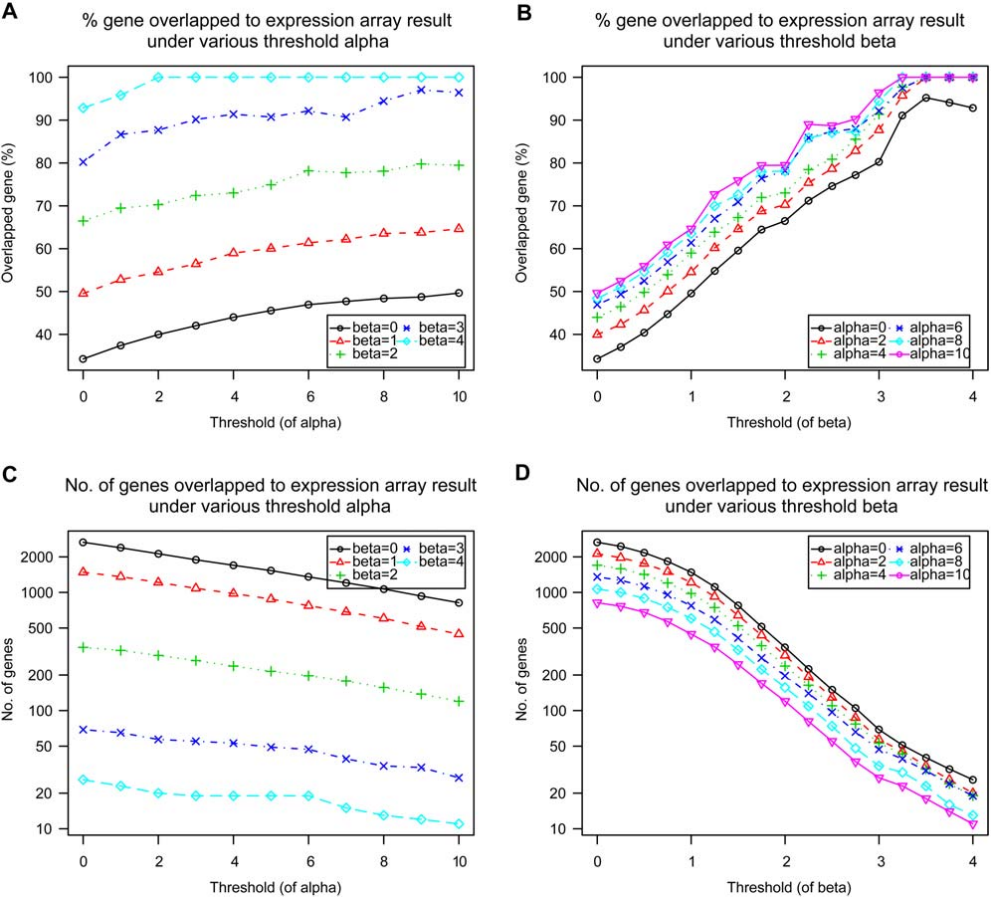
Comparison of tiling array-inferred up-regulated genes and expression array results at various thresholds. The proportion of genes that were identified as up-regulated and also found in the expression array-based gene list at various thresholds of *α* and *β* ((A) and (B)). (C) and (D) represent the number of identified genes.

The threshold setting used to identify regulated genes from the above two integrated tiling array data sets was then examined. In this analysis, genes that only satisfied both *A_hrg_*−*A_control_*≥*α* and *R_hrg_*−*R_control_*≥*β* were compared with results of the expression arrays. Figure 3 represents the percentage (A and B) and number (C and D) of genes that were identified as being up-regulated from the combined tiling array data and which were also found in the expression array results under various threshold (*α* and *β*) settings. As shown, increasing *α* (Figure 3A) and *β* (Figure 3B) yielded higher overlapping rates from the two tiling array data and expression array data sets. In contrast, increasing the threshold resulted in the identification of fewer genes (Figure 3C and D). *β* has a higher contribution to the precision rate and number of genes identified compared with *α*. For specific combinations of *α* and *β* (e.g. *α* = 4 and *β* = 4), 100% of the tiling array inferred that up-regulated genes were found in the expression array results. However, only 19 genes were identified in that setting. There is a trade-off between precision rate and the number of identified genes. Two parameters, *r*_min_ (minimum precision rate) and *N* (number of extracted genes), need to be determined in order to arrive at a reasonable threshold setting. Here, *r*_min_ and *N* were set to 70 and 300, respectively. Under these conditions, the largest *β* value was searched for and α was then determined since a larger threshold results in a higher precision rate. Finally, *α* = 4 and *β* = 2 was obtained and these values were used as thresholds. As a result, 326 and 7 genes were identified as being up- and down-regulated, respectively, following HRG treatment of cells (Table S1). With this setting, the up- and down-regulation of 72.4% of these transcripts (241 out of 333 genes) was confirmed with the expression array data.

### Identification of HRG-induced genes from acetylation and RNA mapping data and confirmation using expression array data

As described above, 326 genes up-regulated following HRG treatment were chosen from the tiling array-based H3K9 acetylation and RNA mapping data sets. Three-hundred and eighteen of the 326 genes (97.5%) have corresponding probe sets on the expression array. The remaining 8 transcripts identified by the tiling array were manually inspected using a web-based genome browser [19]. As a result, only one gene, *LOC400214*, was identified as a novel candidate (Figure S1), which was not mounted on an expression array chip. Although this gene was derived from RefSeq model mRNA (XM_375081), an elevated level of acetylation in the promoter region (2nd and 3rd lanes in Figure S1) and increased RNA map signals in exons (4th and 5th lanes in Figure S1) indicate that this transcript may also be induced following HRG treatment of MCF-7 cells.

Of the 7 down-regulated genes inferred by the tiling array data, genes known to be down-regulated following HRG treatment, including the ER gene (*ESR1*), were not detected in our dataset. This was due to the fact that although the *ESR1* gene was down-regulated as determined by the histone acetylation analysis (56.3 for untreated cells vs. 44.5 for HRG-treated cells), it was slightly up-regulated according to the RNA mapping analysis (8.67 for untreated cells vs. 8.93 for HRG-treated cells). As for the down-regulated genes in our dataset, one gene had no associated probe set in the Affymetrix U133 Plus 2.0 chip and 3 genes were removed due to weak expression intensities. Finally, although the down-regulation of all 3 genes (*ATAD4, C6orf139* and *CRADD*) was confirmed using results of the expression arrays, however, these genes do not possess regulatory binding sites for ER or ERE at the promoter region.

### Relationship between transcriptional activity and copy number

Since MCF-7 is a cancer cell line, there was a concern that high genome copy numbers in the cells may result in strong gene expression in the absence of HRG that may potentially obscure any HRG-related effects. The relationship between gene expression and genome copy number was therefore investigated utilizing the array CGH data. Actively transcribed regions were screened from the whole genome in an unbiased fashion by searching for chromosomal regions with significant acetylation and RNA mapping signals in untreated cells. As a result, 2,163 regions were identified to be actively transcribed in the absence of exogenous stimuli. The average relative copy number in these regions and for the whole genome was 1.12 and 1.03, respectively.

Copy numbers for the 326 HRG-induced up-regulated genes were then examined in an effort to investigate genomic amplifications. The location of genes was obtained from the Entrez Gene database and compared to that of SNP markers where the copy number was calculated. Where a gene locus contained multiple SNP markers, an average copy number was assigned. If no marker was present, copy numbers from the nearest 5′ and 3′ end of a gene were used to calculate the corresponding gene. As a result, an average relative copy number of 1.02 was obtained for the 326 HRG-induced up-regulated genes (1.03 for the whole genome). Seven genes (*GEM*, *KLF10*, *MRPS23*, *MYC*, *NME1*, *PTRH2* and *TRIB1*) had copy numbers greater than 1.5 (Figure S2). However, this low frequency of high copy number in the selected genes can be considered to have negligible contribution to the overall gene expression. Therefore, it was concluded that HRG-induced up-regulation of genes detected by tiling arrays essentially reflects that activity associated with HRG treatment, rather than chromosomal amplification.

### Functional characteristics of HRG-regulated genes

In an effort to investigate the regulatory mechanism relating to the 326 up-regulated and 7 down-regulated genes associated with HRG treatment, the biological function of these genes was examined using two public data sources comprising the transcription factor binding site (TFBS) information in the UCSC genome browser database [20] and the KEGG pathway database [21].

TFBSs for the 333 genes were predicted using the UCSC genome browser database in an effort to provide information on potential upstream regulatory elements that link biological pathways and HRG-regulated gene expression. Genomic coordinate of genes were obtained by mapping the mRNA sequences derived from the UCSC genome browser database. TFBSs located within a 2 kb upstream region from the TSS were then used to find candidate TFs. As a result, 754 potential transcriptional regulatory relationships were found for 62% (208 out of 333) of the HRG-regulated genes. The 208 genes possess predicted TFBSs for 117 TFs (Table S2). Rigorous statistical testing did not result in significant enrichment of the 117 TFs. However, TFs such as *CREB1*, *ATF2* and *EGR1*, which are known to be activated immediately following growth hormone stimulation, showed relatively small *p*-values (*p*<0.05, before multiple test correction).

**Figure 4:**
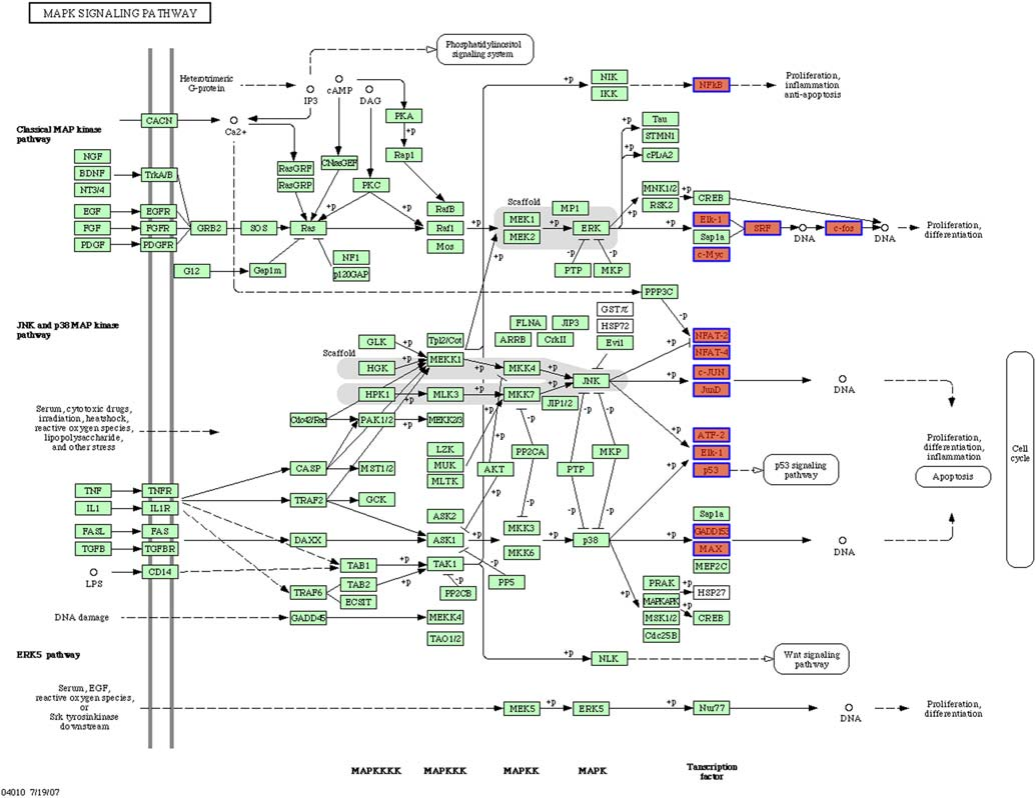
HRG-regulated TFs involved in the KEGG MAPK signaling pathway. Thirteen TFs included in the 117 HRG-regulated TF list involved in the MAPK signaling pathway and are highlighted in red. Note that since *Elk-1* appears twice in the figure (downstream of classical MAP kinase pathway and JNK and p38 MAP kinase pathway), 14 boxes are highlighted.

The predicted TFs were then assigned to signaling pathways using a public database to determine the contribution of specific biological pathways to gene transcription. As a result, the MAPK pathway was found to be the most significant pathway associated with 13 TFs (Figure 4; enrichment of this pathway was supported by Fisher's exact test, *p*<10^−4^). Sixty-nine HRG-regulated genes, which account for 21% of the 333 genes, possess binding sites for 13 MAPK-regulated TFs. Of these, 5 TFs involved in the MAPK pathway possessed small *p*-values (Table S3).

### Relationship with ER and signaling pathway

As shown in Figure 1, HRG treatment facilitated phosphorylation of ER at Ser-118 and Ser-167 and therefore seems to activate ER under the assay conditions used. Whereas E2 did not show Ser-167 phosphorylation of ER in our assay condition, both E2 and HRG are capable of phosphoylating ER at Ser-118 that is required for ER-dependent transcription [22], [23]. Therefore, if a portion of HRG-regulated transcription was mediated by ER in this study, some genes that are also regulated by E2 should be overlapped since E2 and HRG may utilize common ER or ERE-dependent transcription mechanism through Ser-118-mediated ER activation.

A comparison of our gene list with two published gene lists obtained by expression array experiments using E2-stimulated MCF-7 cells [11], [12] was employed to determine whether the 333 HRG-regulated and 239 E2-regulated gene groups contained common genes. As a result, it was found that only 12 genes were common to both gene groups, representing 5.0 and 3.6% of the E2- and HRG-regulated gene groups, respectively. (Table S1).

In an effort to identify upstream regulators common to both HRG- and E2-mediated gene expression, candidate TFs predicted from E2-regulated gene sets were analyzed. TFBS prediction yielded 731 potential transcriptional regulatory relationships that were comprised of 112 TFs and 170 genes for E2. Unexpectedly, of the 239 E2-stimulated genes, only 5 genes (*BHLHB2, BRCA1, DYRK2, FOS* and *GREB1*) possessed binding sites for ER or ERE at the promoter region (within 2 kb upstream from the TSS). This result indicated the potential involvement of transcriptional regulators other than ERE, such as AP-1 and Sp1, in E2-regulated gene expression [24], [25]. A comparison of the 117 HRG TFs and 112 E2 TFs identified a total of 111 TFs common to both TF groups. Furthermore, 12 TFs (10.7% of the 112 TFs) predicted from the E2-regulated genes are involved in the MAPK signaling pathway (Figure S3) and all of which were common to the 13 TFs associated with the HRG-regulated genes. Even when TFs enriched in HRG- and E2-regulated genes were investigated (*p*<0.05, before multiple test correction), 4 TFs were still identified as being common to the 14 and 13 HRG- and E2-related TF groups, respectively. For example, *ARNT* was commonly predicted from 9 HRG-regulated and 7 E2-regulated genes. Interestingly, however, the expression level of *KLF6,* one of the target genes of *ARNT*, increased with HRG treatment and decreased with E2 treatment. This result indicates that TFs activated following E2 and HRG stimulation may yield opposite effects in terms of gene regulation. Additionally, our analysis revealed that HRG- and E2-regulated gene sets contain few common members. These overall analytical results suggest that E2 and HRG depend considerably on the MAPK pathway for transcriptional control, where each TF may be differentially regulated by other factors, thereby resulting in the induction of different sets of genes. However, as expected, the presence of enriched TFs in the MAPK signaling pathway, 5 (out of 14) TFs for HRG and 1 (out of 13) TFs for E2, suggests that HRG is more dependent on this pathway.

## Discussion

In this study, integrative analysis of HRG-regulated gene expression in MCF-7 cells was performed using two types of tiling array data, comprising histone H3K9 acetylation and RNA mapping, and compared the results with those based on expression array and array CGH. Following the determination of various threshold settings, 333 transcripts that were regulated by HRG were finally identified. The up- and down-regulation of 72.4% of these transcripts (241 of the 333 genes) was confirmed using expression arrays.

EGF, a growth hormone ligand for EGFR/ErbB1 receptor, is known to induce coupling of phosphorylation and acetylation of histone H3 that results in enhanced gene expression [26]. Our current analysis with HRG also indicated that histone acetylation and gene up-regulation may be closely related.

**Figure 5:**
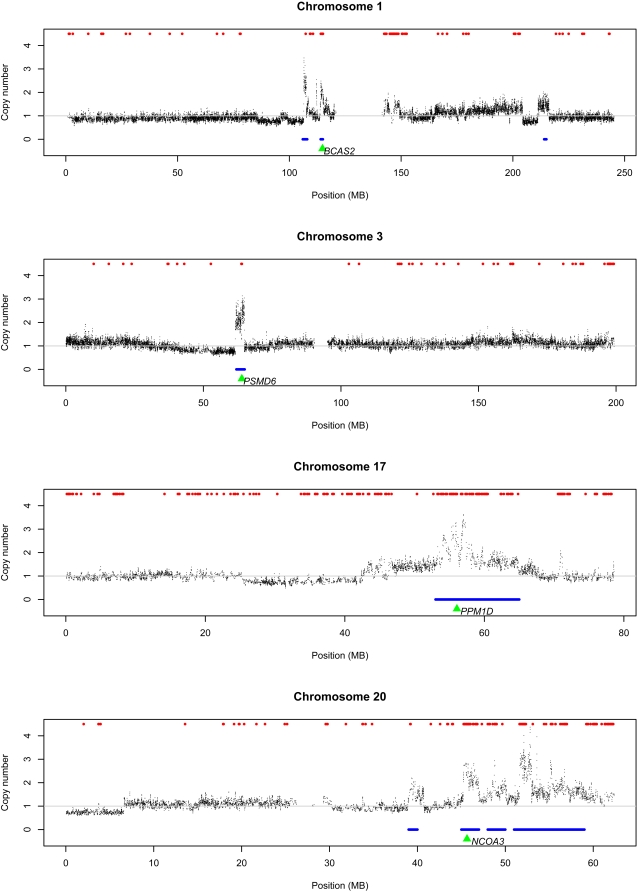
Chromosome-wide plots for copy number distribution. Four representative chromosomes (1, 3, 17 and 20) are shown in this figure. See Figure S2 for the same plots for all chromosomes. Red and blue bars at the top and bottom in each plot designate actively transcribed and high copy number regions, respectively. Green triangles represent location of the *BCAS2* (chromosome 1), *PSMD6* (chromosome 3) , *PPM1D* (chromosome 17) and *NCOA3* (chromosome 20) gene loci, respectively.

**Figure 6:**
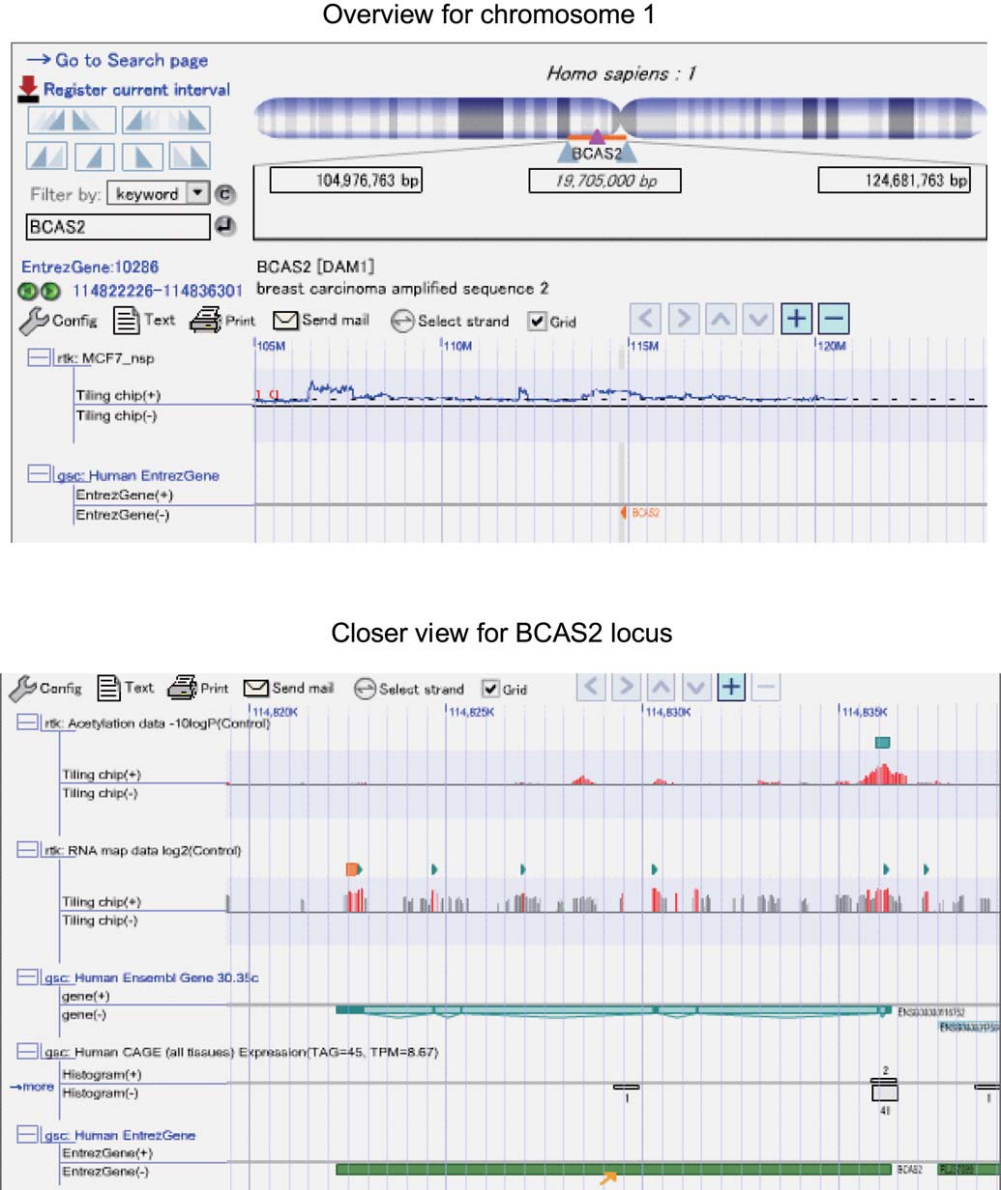
OmicBrowse display for the *BCAS2* gene. Experimental results (tiling arrays and array CGH) together with genome annotation are represented along the genomic coordinate using OmicBrowse, a web-based genome browser. The upper panel shows the copy number (1st lane) and *BCAS2* gene locus (2nd lane) in chromosome 1. The lower panel represents a detailed view of this locus with 5 lanes. Each lane represents 1) H3K9 acetylation signals in the control experiment, 2) RNA mapping signal in the control experiment, 3) Ensembl Gene with exon-intron structure, 4) human CAGE tag counts, and 5) a region of *BCAS2* derived from the NCBI Entrez Gene database. The height of the vertical bars in the 1st and 2nd lanes corresponds to the strength of the signals. Light green bars in the top 2 lanes represent regions with significant signal intensity. The upper panel shows that *BCAS2* is located in amplified regions. In the lower panel, a high level of acetylation around the TSS, significant and higher signal intensity of the RNA map at exons, and a high CAGE tag can be seen for *BCAS2*. These data support the view that *BCAS2* was expressed in the absence of exogenous stimuli.

Our current results indicated that the majority of HRG-regulated genes were up-regulated (326 of the 333 genes). However, since MCF-7 is a cancer cell line, there was a concern that high genome copy numbers in the cells may result in strong gene expression in the absence of HRG that may potentially obscure any HGR-related effects. Genes such as *NCOA3*, *PPM1D*, *PSMD6* and *BCAS2* were detected with high expression and copy numbers in MCF-7 cells (Figures 5 and 6), and these genes have been associated with breast cancer progression [27]–[30]. However, our analysis revealed that the overall correlation between average copy number and the extent of gene expression was surprisingly weak.

The frequency of occurrence of ER or ERE-mediated transcription was investigated using integrated gene expression analysis. Surprisingly, whereas HRG treatment resulted in the phosphorylation of ER at Ser-118, which is thought to lead to ER activation, ER- or ERE-dependent transcription induced by HRG seemed to be quite minor (0.6%). To account for this observation we initially speculated that it was due to the short exposure time (2 hrs) of the cells to HRG, or perhaps to a lack of depletion of E2-like compounds from the culture medium, which are thought to down-regulate ER levels [15], [31]. In an effort to address this issue, it was confirmed by western blot analysis that HRG could phosphorylate ER under the culture conditions used (Figure 1). As for the exposure time, given that 4–48 hrs of E2-stimulated gene regulation failed to show ERE at its upstream region, a complex regulatory mechanism for gene expression rather than the exposure time of cells to stimuli seems to be a major factor. In fact, recent analysis of ER-alpha binding on the whole genome revealed that 95% of ERE binding sites of targeted genes are located at intronic or distal locations (>5 kb from 5′ and 3′ ends of the transcript) [32], its regulation seems to be far more complex than that associated with MAPK-regulated transcription. Additionally, our current analytical results suggested the presence of distinct regulatory roles for TFs that yield opposite effects in terms of HRG- and E2-induced gene regulation. ER expression and activation is widely regulated by various signaling pathways and kinases [33]–[35]. The current study has provided some insight into the similarities and differences between complex regulatory mechanisms in growth hormone- and E2-induced gene expression in ER-positive breast cancer cells.

## Materials and Methods

### Cell culture

The MCF-7 human breast cancer cell line was obtained from the American Type Culture Collection and maintained in DMEM medium supplemented with 10% fetal bovine serum. Prior to growth hormone treatment, cells were serum-starved for 16–24 hours. For the expression analysis, 10 nM HRG-β 176–246 (R&D Systems) was added to the cells and incubated for 2 hrs. Cells untreated with growth hormone were used as the control.

### ER phosphorylation

For the ER phosphorylation assay, cells were incubated with HRG (10 nM) or E2 (10 nM) for up to 90 min. Cells were then rinsed with ice-cold PBS and lysed using the Bio-Plex Cell Lysis Kit (Bio-Rad Laboratories). The cell lysate was cleared by centrifugation, and the protein concentration of the supernatant was determined using DC protein assay reagent (Bio-Rad Laboratories). Equal amounts of protein were subjected to Western blot analysis. Electrophoretically resolved proteins were blotted and subsequently probed with anti-phospho-Ser-118 (Upstate Biotechnology, Inc.), anti-phospho-Ser-167 (Cell Signaling Technology, Inc.) and anti-ER (Upstate Biotechnology, Inc.) antibodies. Phosphorylated proteins were quantified using a densitometer.

### Expression array

Expression array experiments were performed using the Affymetrix Human Genome U133 Plus 2.0 Chip (Affymetrix, Santa Clara, CA, USA) that encodes 54,000 probe sets (with 47,000 transcripts and variants that includes 38,500 well-characterized genes) for integrative expression analyses of RNA mapping and histone acetylation, and for verification of the expression of novel transcripts. Hybridization and washing were performed according to the manufacturer's protocol. Signals were processed according to the GeneChip expression analysis algorithm (GCOS ver. 1.2) (Affymetrix). Probe sets with a low signal (100) were discarded in subsequent analyses. Relative changes in expression levels of HRG-treated cells against untreated cells were transformed using a log2 function. A gene was regarded as being up-regulated if this value was greater than 0.

### ChIP on chip experiment to measure the level of acetylation at lysine 9 of histone H3

The level of enrichment for acetylation at lysine 9 of histone H3 (H3K9Ac) in the human genome was determined using the Affymetrix whole-genome tiling array (Affymetrix Human tiling array 1.0), which tiles the non-repetitive portion of the human genome at 35-bp intervals with more than 41 million pairs of 25-mer probe sequences. The experimental protocol employed was that previously described [36]. MCF-7 cells were fixed with 1% formaldehyde and sonicated using a Branson 450 Sonifier. The sonicated lysates were pre-cleared using protein G sepharose. Following removal of control aliquots, the pre-cleared lysates were treated with anti-acetylated K9 histone H3 antibody (Upstate Biotechnology, Inc.). The immune complexes were precipitated with protein G dynabeads (Dynal, Invitrogen), washed and eluted from the beads. The eluted immune complexes were reverse cross-linked and treated with RNase and Proteinase K. The precipitated DNA was extracted using a phenol/chloroform method and ethanol precipitation. Both the precipitated (treated) and control DNA (whole-cell extract) was amplified using the ligation mediated (LM)-PCR method. The amplified DNA was purified, fragmented with DNase I and end-labeled with biotin-ddATP. The end-labeled DNA was hybridized to the tiling array for 18 hrs at 45°C. Following hybridization, arrays were washed and scanned using the Affymetrix GeneChip System.

The hybridization intensities (background-subtracted intensity; PM–MM, where PM and MM represent intensities detected by a 25-mer comprising perfectly matching and one-base-mismatching genome sequences, respectively) of the probes were measured in triplicate for each of the treated and control samples. A shift in intensity of the treated relative to control data in a 400-bp window centered at each probe was evaluated using a Wilcoxon Rank Sum test, which assigned a *p*-value to the probe position [37]. The TAS Affymetrix software (http://www.affymetrix.com/support/developer/downloads/TilingArrayTools) was used for *p*-value calculations.

### RNA mapping and computation of signal intensity

RNA mapping experiments using the Affymetrix tiling array were performed as previously described [38]. Fifteen micrograms of total RNA was used as a template for first-strand synthesis. Double-stranded cDNAs were synthesized using reverse transcribed cDNAs as templates. Double-stranded cDNAs were purified, fragmented, end-labeled and hybridized to the tiling arrays in triplicate. Hybridized arrays were washed and scanned using the Affymetrix GeneChip System. The signal intensity at each probe position was computed using the TAS Affymetrix software. A Wilcoxon signed-rank test was applied to hybridization intensities measured at probes located within ±50 bp of every probe location, and the pseudomedian generated by the Wilcoxon test was assigned as an estimate of the signal intensity to the probe position [39]. The signal intensity is given on a log2 scale.

### Level of acetylation at lysine 9 of histone H3 in the promoter region of protein-coding genes

Using the 5′ ends of mRNA sequences (RefSeq database: ftp://ftp.ncbi.nih.gov/refseq) aligned to the genome as the TSS, we attempted to measure the magnitude of H3K9Ac enrichment in the promoter region (±1.5 kb of TSS) of the genes. We considered the highest, average and median of −10 log (*p*-value) detected by the probes located in the promoter region.

### Estimation of the expression level of genes from the RNA mapping data

The signal intensities given by the RNA mapping were used to estimate the expression level of genes in the human genome. The average signal intensity of the probes located in exons of each gene was used as an indicator of the expression level. Only genes that were mapped by 50 (tentative) or more probes were used in the analysis.

### Array CGH analysis

Genome-wide DNA screening on an Affymetrix GeneChip Mapping 250K Nsp EA array was performed using the GeneChip Instrument system according to the standard protocols of the manufacturer (Affymetrix). Briefly, genomic DNA was digested with restriction endonuclease, ligated to an adaptor, and subjected to PCR amplification with adaptor-specific primers. The PCR products were digested with DNase I and labeled with a biotinylated nucleotide analogue using terminal deoxynucleotidyl transferase. The labeled DNA fragments were hybridized to the microarray, the hybridized DNA probes were captured by streptavidin-phycoerythrin conjugates, and the array was scanned. The signal intensity ratio was calculated as previously described with some modifications [40]. For this analysis, the publicly available data-Gene Expression Omnibus (http://www.ncbi.nlm.nih.gov/geo/) accession number GSE5013 was employed as the reference data. A moving average of the signal intensity ratio across 5 adjacent markers was calculated and mapped onto the human genome according to the physical position of each marker. To calculate signal intensity ratio for X chromosome, gender information of the sample is necessary. Because called gender is not reported in the report file for Affymetrix GeneChip Mapping 250K Nsp EA array, we did not calculate signal intensity ratio for X chromosome in this study.

### Identification of HRG-induced up- and down-regulated genes from the H3K9 acetylation and RNA mapping data

In an effort to identify HRG-induced up-regulated genes, signal intensities in the control experiment (without HRG treatment) were subtracted from those obtained following HRG treatment. Genes satisfying both *A_hrg_*−*A_control_*≥*α* and *R_hrg_*−*R_control_*≥*β* were regarded as being up-regulated. Here, *A_control_* and *A_hrg_* represent the acetylation signal (average of −10 log (*p*-value) in the promoter region) before and after HRG treatment, respectively. Similarly, *R_control_* and *R_hrg_* refer to the average RNA map signals in exons in untreated cells and 2 hrs after HRG treatment, respectively. For down-regulated genes, the left-hand member was multiplied by -1. Thresholds *α* and *β* were set to 4 and 2, respectively. After obtaining the list of RefSeq accession numbers, these were converted to Entrez Gene IDs using the accession number mapping table provided at the NCBI ftp site (ftp://ftp.ncbi.nih.gov/gene/DATA). RefSeq entries without corresponding Entrez Gene IDs were discarded in subsequent analyses.

### Prediction of upstream TF binding site

In an effort to determine potential transcriptional regulatory relationships, TFBS predicted from the UCSC genome browser database (http://hgdownload.cse.ucsc.edu/goldenPath/hg17/database) were utilized. Since the version of the human genome used in the tiling array and array CGH analysis was NCBI build 35, we used a corresponding data set (hg17). The region corresponding to the binding site sequence search was restricted to a 2 kb upstream region from the TSS. The genomic location of mRNA was downloaded from the UCSC genome browser database and the first base was assigned as the TSS. The relation between the mRNA accession numbers and Entrez Gene IDs was determined from the mapping table provided at the NCBI ftp site (ftp://ftp.ncbi.nih.gov/gene/DATA). TF Gene IDs were initially searched for using UniProt accession numbers. When no information was found, TF names were matched against gene symbols. When no Gene ID was found, the TF was discarded from the analysis. Enrichment of TFBSs for a specific gene set was tested using Fisher's exact test (implemented using the ‘fisher.test’ function in the R language) followed by Bonferroni's correction. When searching for ER binding sites, in addition to predicted TFBSs as described above, information from TRANSFAC [41] and pattern search implementation using 3 consensus sequences, TCGACGCTTTCAAGGTCATATCCG, TCGACAAAGTCAGGTCACAGTGACCTGATCAAG and GGTCANNNTGACC, where ‘N’ represents an arbitrary nucleotide, [9], [42] were also utilized.

## Supporting Information

Figure S1 OmicBrowse display for the *LOC400214* gene. The OmicBrowse display comprises 8 lanes and each lane represents 1) copy numbers in non-treated cells, 2) and 3) H3K9 acetylation signals in the control and HRG-treated cells, respectively, 4) and 5) RNA mapping signals in the control and HRG-treated cells, respectively, 6) location of Ensembl Gene with exon-intron structure, 7) human CAGE tag counts, and 8) location of the gene obtained from NCBI Entrez Gene. The height of the vertical bars in the 2nd, 3rd, 4th and 5th lanes reflects the strength of the signals. Light green vertical bars in these lanes show regions with significant signal intensity.
(2.86 MB TIF)

Figure S2 Copy number distribution in MCF-7 cells. Red and blue bars at the top and bottom in each plot designate actively transcribed and high copy number regions, respectively. The location of 7 genes (*GEM, KLF10, MRPS23, MYC, NME1, PTRH2* and *TRIB1*) is represented by green triangles.
(1.21 MB PDF)

Figure S3 E2-regulated TFs involved in the KEGG MAPK signaling pathway. Twelve TFs related to estrogen-induced genes involved in the KEGG MAPK signaling pathway are highlighted by a blue box. TFs are color-coded according to changes in expression of target genes (blue: down, purple: both up and down). Note that since *Elk-1* appears twice in the figure (downstream of classical MAP kinase pathway and JNK and p38 MAP kinase pathway), 13 boxes are highlighted.
(2.84 MB TIF)

Table S1 HRG-induced up- and down-regulated genes identified from integrated H3K9 acetylation and RNA mapping data
(0.07 MB XLS)

Table S2 The list of predicted transcription factors for HRG-induced up- and down-regulated genes
(0.02 MB XLS)

Table S3 The list of predicted transcription factors with relatively small p-values for HRG- and E2-induced genes.
(0.02 MB XLS)

## References

[1] Weiss FU, Wallasch C, Campiglio M, Issing W, Ullrich A (1997). Distinct characteristics of heregulin signals mediated by HER3 or HER4.. J Cell Physiol.

[2] Carraway KL, Soltoff SP, Diamonti AJ, Cantley LC (1995). Heregulin stimulates mitogenesis and phosphatidylinositol 3-kinase in mouse fibroblasts transfected with erbB2/neu and erbB3.. J Biol Chem.

[3] Tan M, Grijalva R, Yu D (1999). Heregulin beta1-activated phosphatidylinositol 3-kinase enhances aggregation of MCF-7 breast cancer cells independent of extracellular signal-regulated kinase. Cancer Res.

[4] Nagashima T, Shimodaira H, Ide K, Nakakuki T, Tani Y (2007). Quantitative transcriptional control of ErbB receptor signaling undergoes graded to biphasic response for cell differentiation.. J Biol Chem.

[5] Yarden Y, Sliwkowski MX (2001). Untangling the ErbB signalling network.. Nat Rev Mol Cell Biol..

[6] Bunone G, Briand PA, Miksicek RJ, Picard D (1996). Activation of the unliganded estrogen receptor by EGF involves the MAP kinase pathway and direct phosphorylation.. EMBO J.

[7] Campbell RA, Bhat-Nakshatri P, Patel NM, Constantinidou D, Ali S, Nakshatri H (2001). Phosphatidylinositol 3-kinase/AKT-mediated activation of estrogen receptor alpha: a new model for anti-estrogen resistance.. J Biol Chem.

[8] Martin MB, Franke TF, Stoica GE, Chambon P, Katzenellenbogen BS (2000). A role for Akt in mediating the estrogenic functions of epidermal growth factor and insulin-like growth factor I.. Endocrinology.

[9] Klinge CM (2001). Estrogen receptor interaction with estrogen response elements.. Nucleic Acids Res.

[10] Mazumdar A, Wang RA, Mishra SK, Adam L, Bagheri-Yarmand R (2001). Transcriptional repression of oestrogen receptor by metastasis-associated protein 1 corepressor.. Nat Cell Biol.

[11] Frasor J, Stossi F, Danes JM, Komm B, Lyttle CR (2004). Selective estrogen receptor modulators: discrimination of agonistic versus antagonistic activities by gene expression profiling in breast cancer cells.. Cancer Res.

[12] Frasor J, Danes JM, Komm B, Chang KC, Lyttle CR (2003). Profiling of estrogen up- and down-regulated gene expression in human breast cancer cells: insights into gene networks and pathways underlying estrogenic control of proliferation and cell phenotype.. Endocrinology.

[13] Kumar V, Chambon P (1998). The estrogen receptor binds tightly to its responsive element as a ligand-induced homodimer.. Cell.

[14] Joel PB, Smith J, Sturgill TW, Fisher TL, Blenis J (1998). pp90rsk1 regulates estrogen receptor-mediated transcription through phosphorylation of Ser-167. Mol Cell Biol..

[15] Pietras RJ, Arboleda J, Reese DM, Wongvipat N, Pegram MD (1995). HER-2 tyrosine kinase pathway targets estrogen receptor and promotes hormone-independent growth in human breast cancer cells.. Oncogene.

[16] Shahbazian MD, Grunstein M (2007). Functions of Site-Specific Histone Acetylation and Deacetylation.. Annu Rev Biochem.

[17] Fann M, Godlove JM, Catalfamo M, Wood WH, Chrest FJ (2006). Histone acetylation is associated with differential gene expression in the rapid and robust memory CD8(+) T-cell response.. Blood.

[18] Bertone P, Stolc V, Royce TE, Rozowsky JS, Urban AE (2004). Global identification of human transcribed sequences with genome tiling arrays.. Science.

[19] Toyoda T, Mochizuki Y, Player K, Heida N, Kobayashi N (2007). OmicBrowse: a browser of multidimensional omics annotations.. Bioinformatics.

[20] Karolchik D, Baertsch R, Diekhans M, Furey TS, Hinrichs A (2003). The UCSC Genome Browser Database.. Nucleic Acids Res.

[21] Kanehisa M, Goto S, Hattori M, Aoki-Kinoshita KF, Itoh M (2006). From genomics to chemical genomics: new developments in KEGG.. Nucleic Acids Res.

[22] Ali S, Metzger D, Bornert JM, Chambon P (1993). Modulation of transcriptional activation by ligand-dependent phosphorylation of the human oestrogen receptor A/B region.. EMBO J.

[23] Cheng J, Zhang C, Shapiro DJ (2007). A functional serine 118 phosphorylation site in estrogen receptor-alpha is required for down-regulation of gene expression by 17beta-estradiol and 4-hydroxytamoxifen.. Endocrinology.

[24] Moggs JG, Orphanides G (2001). Estrogen receptors: orchestrators of pleiotropic cellular responses.. EMBO Rep.

[25] Nilsson S, Makela S, Treuter E, Tujague M, Thomsen J (2001). Mechanisms of estrogen action.. Physiol Rev.

[26] Cheung P, Tanner KG, Cheung WL, Sassone-Corsi P, Denu JM (2000). Synergistic coupling of histone H3 phosphorylation and acetylation in response to epidermal growth factor stimulation.. Mol Cell.

[27] Torres-Arzayus MI, Font de Mora J, Yuan J, Vazquez F, Bronson R (2004). High tumor incidence and activation of the PI3K/AKT pathway in transgenic mice define AIB1 as an oncogene.. Cancer Cell.

[28] Proia DA, Nannenga BW, Donehower LA, Weigel NL (2006). Dual roles for the phosphatase PPM1D in regulating progesterone receptor function.. J Biol Chem.

[29] Ren S, Smith MJ, Louro ID, McKie-Bell P, Bani MR (2000). The p44S10 locus, encoding a subunit of the proteasome regulatory particle, is amplified during progression of cutaneous malignant melanoma.. Oncogene.

[30] Maass N, Rosel F, Schem C, Hitomi J, Jonat W (2002). Amplification of the BCAS2 gene at chromosome 1p13.3-21 in human primary breast cancer.. Cancer Lett.

[31] Grunt TW, Saceda M, Martin MB, Lupu R, Dittrich E (1995). Bidirectional interactions between the estrogen receptor and the cerbB-2 signaling pathways: heregulin inhibits estrogenic effects in breast cancer cells.. Int J Cancer.

[32] Lin CY, Vega VB, Thomsen JS, Zhang T, Kong SL, Xie M (2007). Whole-genome cartography of estrogen receptor alpha binding sites.. PLoS Genet.

[33] Arnold SF, Obourn JD, Jaffe H, Notides AC (1995). Phosphorylation of the human estrogen receptor by mitogen-activated protein kinase and casein kinase II: consequence on DNA binding.. J Steroid Biochem Mol Biol.

[34] Simoncini T, Hafezi-Moghadam A, Brazil DP, Ley K, Chin WW (2000). Interaction of oestrogen receptor with the regulatory subunit of phosphatidylinositol-3-OH kinase.. Nature.

[35] Driggers PH, Segars JH (2002). Estrogen action and cytoplasmic signaling pathways. Part II: the role of growth factors and phosphorylation in estrogen signaling.. Trends Endocrinol Metab.

[36] Nishida H, Suzuki T, Kondo S, Miura H, Fujimura F (2006). Histone H3 acetylated at lysine 9 in promoter is associated with low nucleosome density in the vicinity of transcription start site in human cell.. Chromosome Res.

[37] Bernstein BE, Kamal M, Lindblad-Toh K, Bekiranov S, Bailey DK (2005). Genomic maps and comparative analysis of histone modifications in human and mouse.. Cell.

[38] Hasegawa Y, Fukuda S, Shimokawa K, Kondo S, Maeda N (2006). A RecA-mediated exon profiling method.. Nucleic Acids Res.

[39] Kampa D, Cheng J, Kapranov P, Yamanaka M, Brubaker S (2004). Novel RNAs identified an in-depth analysis of the transcriptome of human chromosomes 21 and 22.. Genome Res.

[40] Kojima T, Mukai W, Fuma D, Ueda Y, Okada M (2006). Determination of genomic breakpoints in an epileptic patient using genotyping array.. Biochem Biophys Res Commun.

[41] Matys V, Kel-Margoulis OV, Fricke E, Liebich I, Land S (2006). TRANSFAC and its module TRANSCompel: transcriptional gene regulation in eukaryotes.. Nucleic Acids Res.

[42] Barry JB, Giguere V (2005). Epidermal growth factor-induced signaling in breast cancer cells results in selective target gene activation by orphan nuclear receptor estrogen-related receptor alpha.. Cancer Res.

