# Supplementary figures and images for: Integrative Genome-Wide Expression Analysis Bears Evidence of Estrogen Receptor-Independent Transcription in Heregulin-Stimulated MCF-7 Cells
Source: PLoS One. 2008 Mar 19;3(3):e1803. doi: 10.1371/journal.pone.0001803 (PMC2266794; doi:10.1371/journal.pone.0001803)

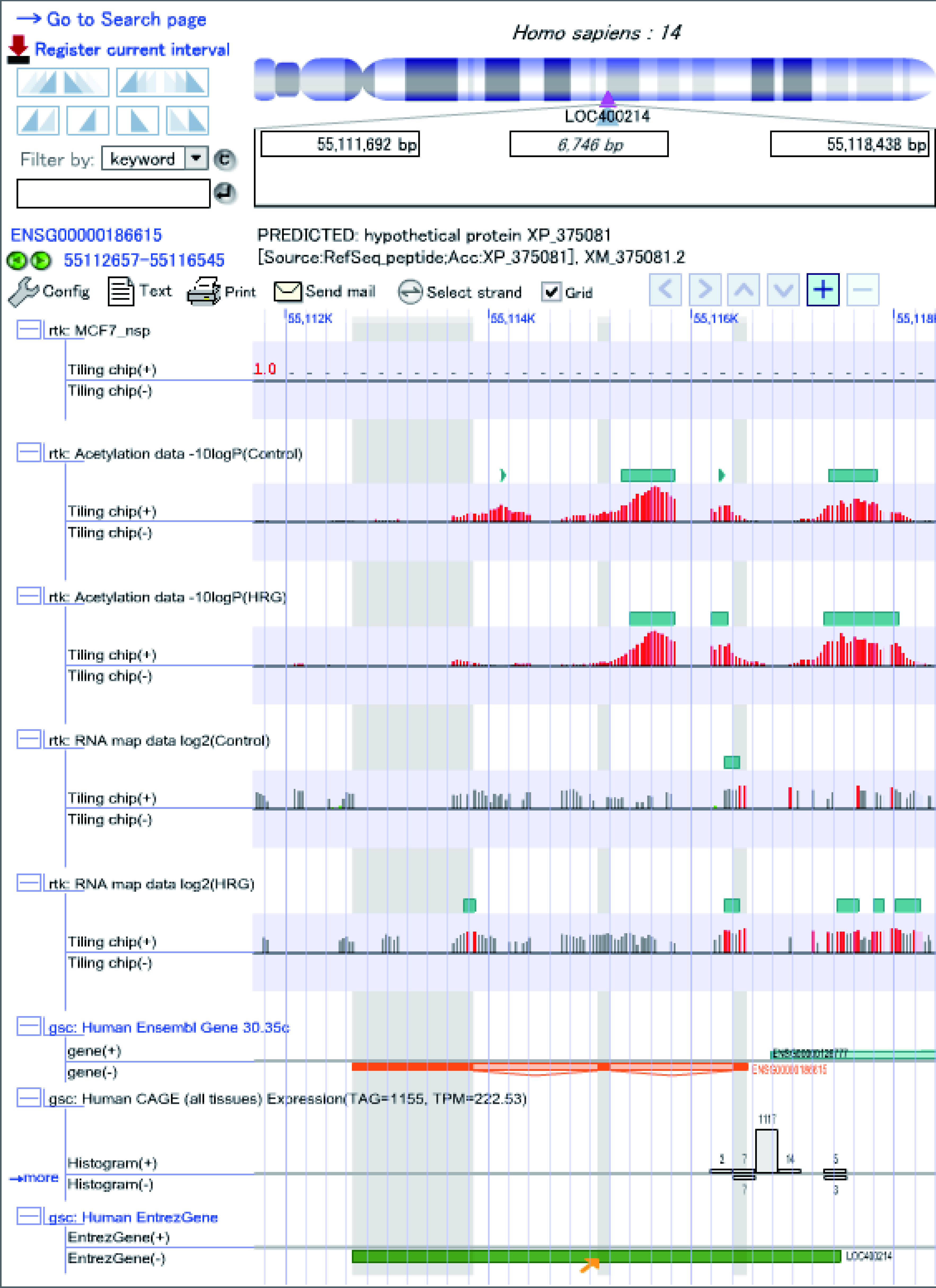

Supplement: Figure S1 — OmicBrowse display for the LOC400214 gene. The OmicBrowse display comprises 8 lanes and each lane represents 1) copy numbers in non-treated cells, 2) and 3) H3K9 acetylation signals in the control and HRG-treated cells, respectively, 4) and 5) RNA mapping signals in the control and HRG-treated cells, respectively, 6) location of Ensembl Gene with exon-intron structure, 7) human CAGE tag counts, and 8) location of the gene obtained from NCBI Entrez Gene. The height of the vertical bars in the 2nd, 3rd, 4th and 5th lanes reflects the strength of the signals. Light green vertical bars in these lanes show regions with significant signal intensity. (2.86 MB TIF) [file pone.0001803.s001.tif]

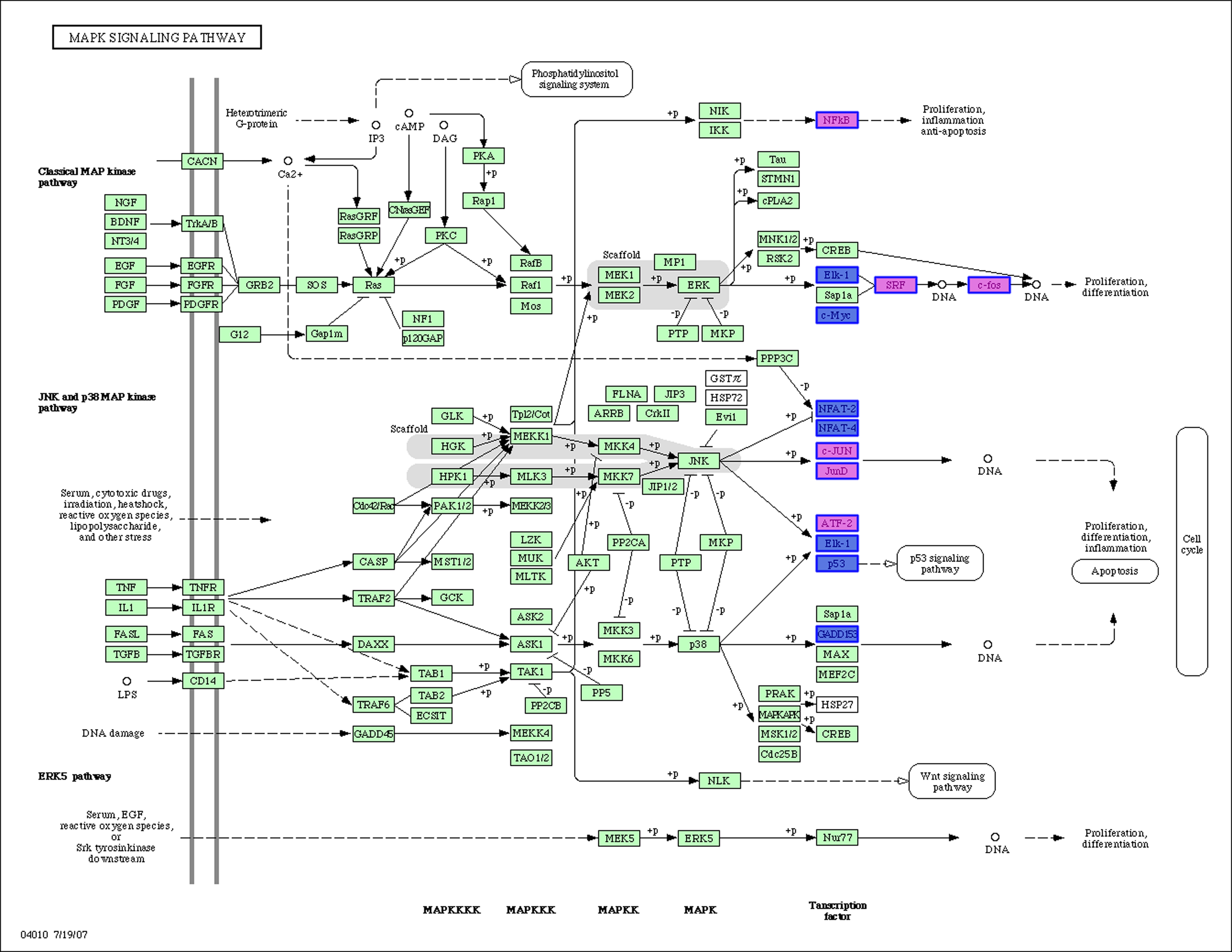

Supplement: Figure S3 — E2-regulated TFs involved in the KEGG MAPK signaling pathway. Twelve TFs related to estrogen-induced genes involved in the KEGG MAPK signaling pathway are highlighted by a blue box. TFs are color-coded according to changes in expression of target genes (blue: down, purple: both up and down). Note that since Elk-1 appears twice in the figure (downstream of classical MAP kinase pathway and JNK and p38 MAP kinase pathway), 13 boxes are highlighted. (2.84 MB TIF) [file pone.0001803.s003.tif]
