# Supplementary material for: Integrative Genome-Wide Expression Analysis Bears Evidence of Estrogen Receptor-Independent Transcription in Heregulin-Stimulated MCF-7 Cells
Source: PLoS One. 2008 Mar 19;3(3):e1803. doi: 10.1371/journal.pone.0001803 (PMC2266794; doi:10.1371/journal.pone.0001803)
Supplement: Figure S2 — Copy number distribution in MCF-7 cells. Red and blue bars at the top and bottom in each plot designate actively transcribed and high copy number regions, respectively. The location of 7 genes (GEM, KLF10, MRPS23, MYC, NME1, PTRH2 and TRIB1) is represented by green triangles. (1.21 MB PDF) [file pone.0001803.s002.pdf]

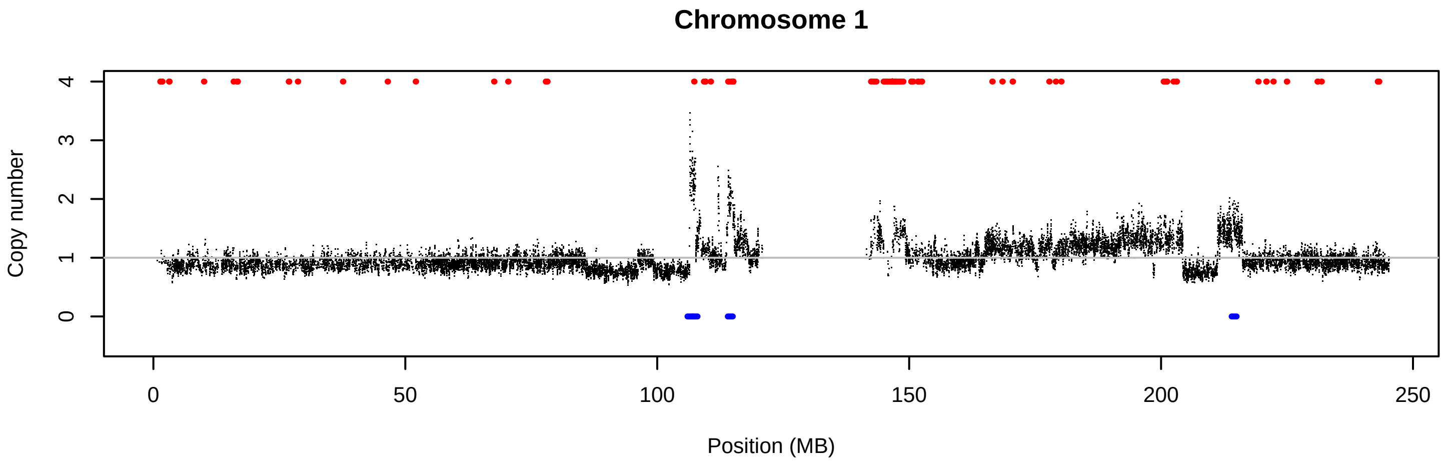
Chromosome 1
Copy number
0
1
2
3
4
0
50
100
150
200
250
Position (MB)

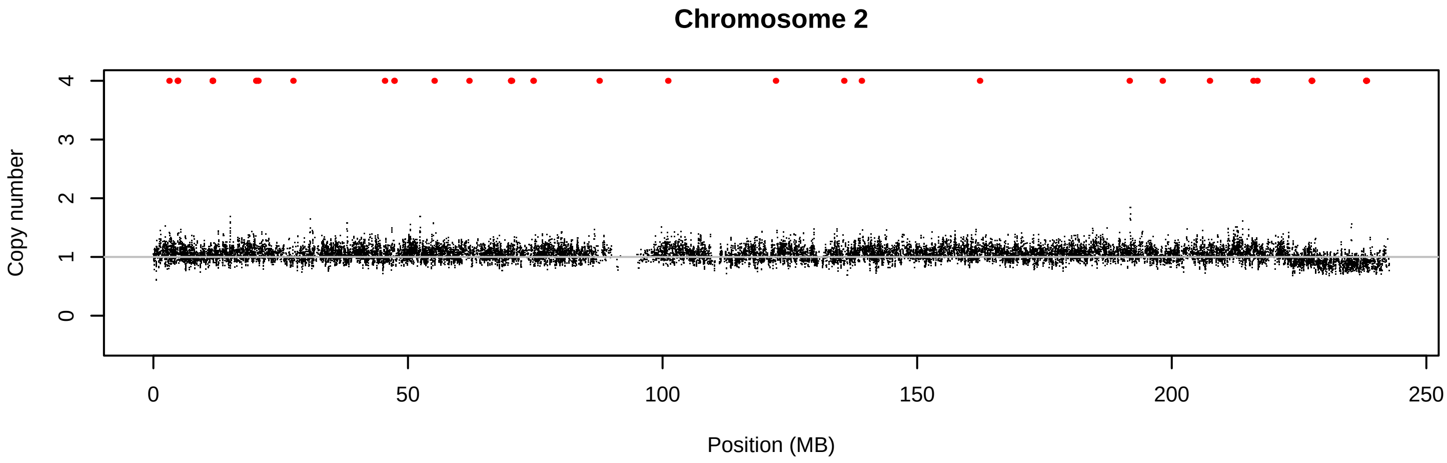
Chromosome 2
Copy number
0
1
2
3
4
0
50
100
150
200
250
Position (MB)

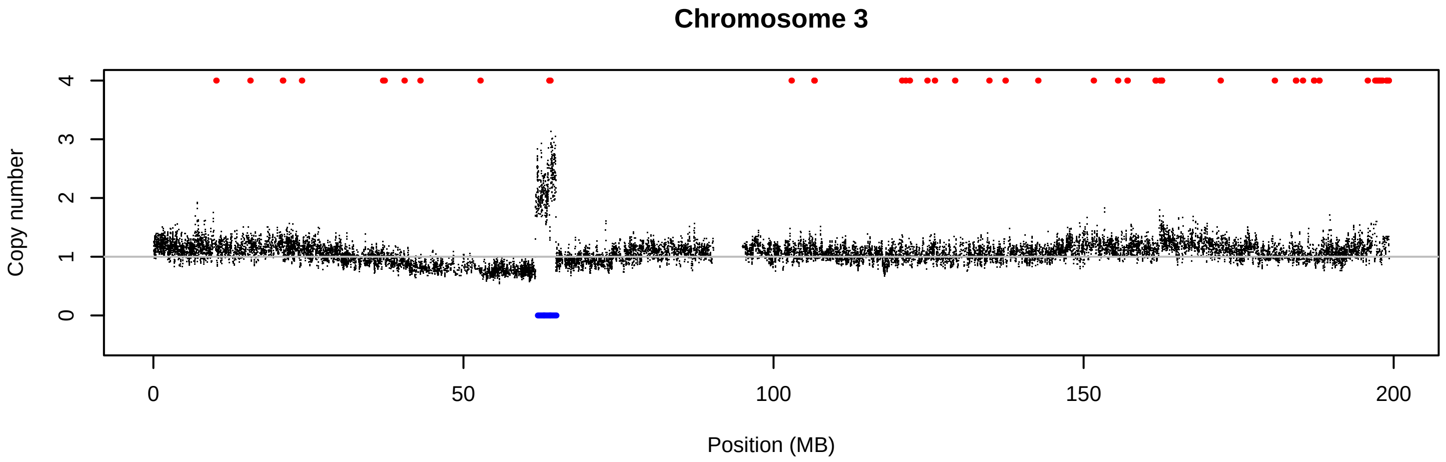
Chromosome 3
Copy number
0
1
2
3
4
0
50
100
150
200
Position (MB)

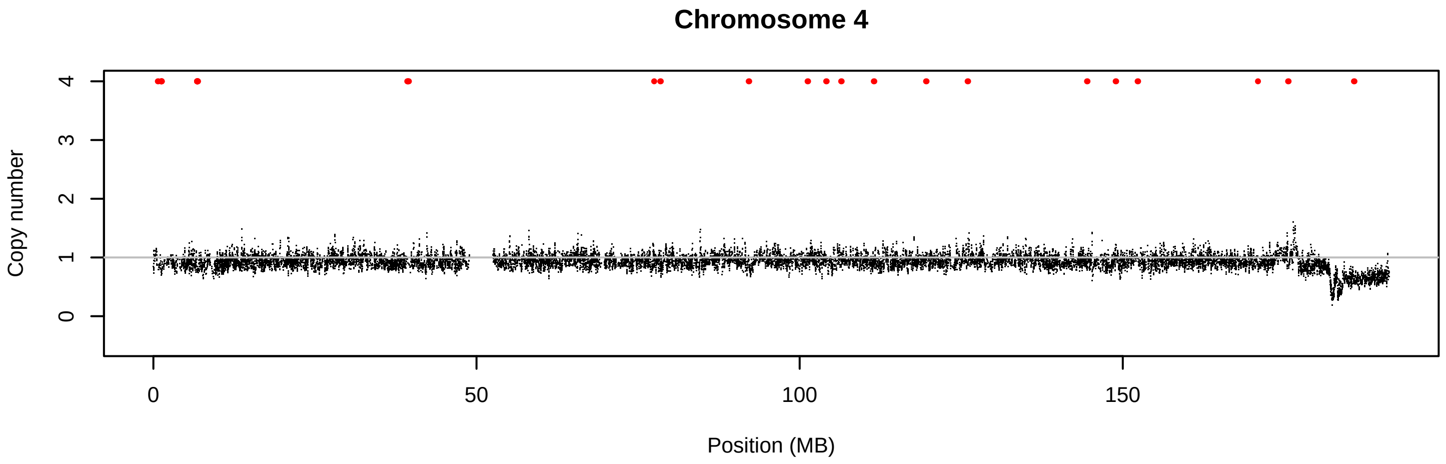
Chromosome 4
Copy number
0
1
2
3
4
0
50
100
150
Position (MB)

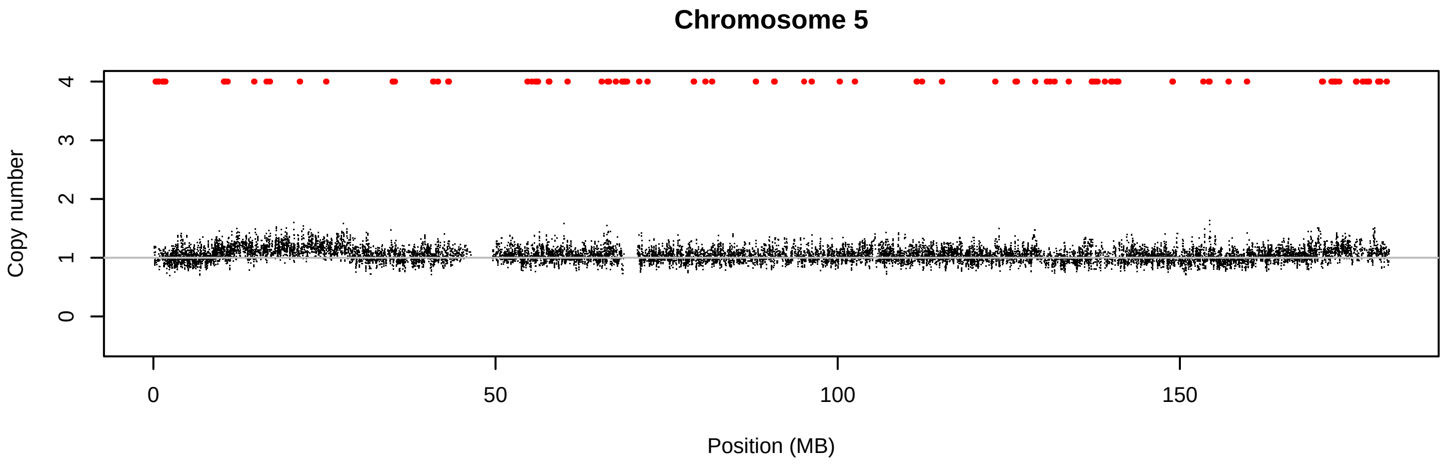
Chromosome 5
Copy number
0
1
2
3
4
0
50
100
150
Position (MB)

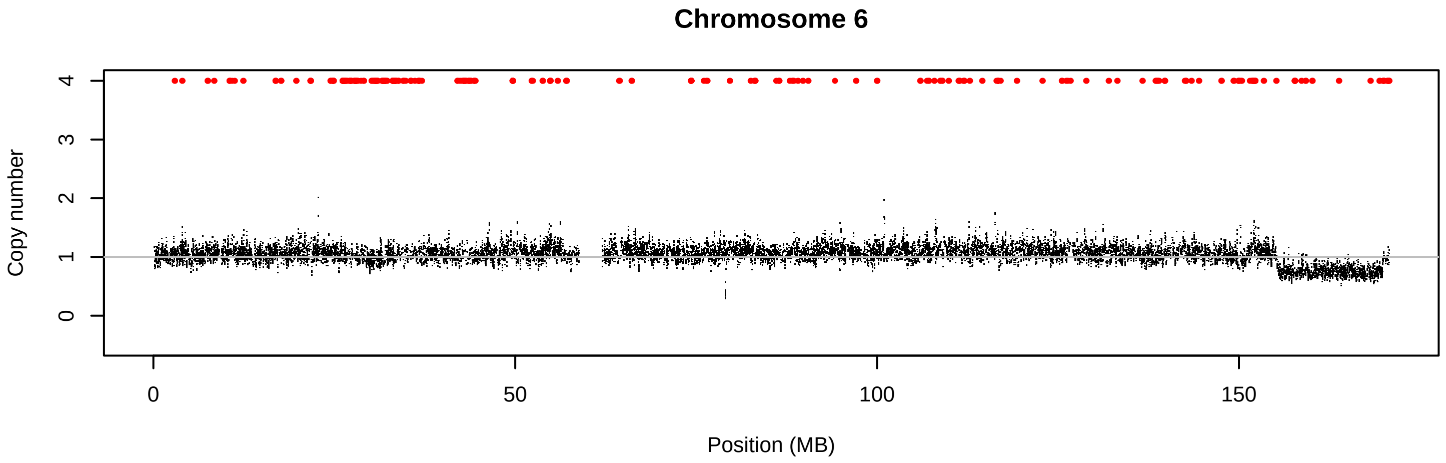
Chromosome 6
Copy number
0
1
2
3
4
0
50
100
150
Position (MB)

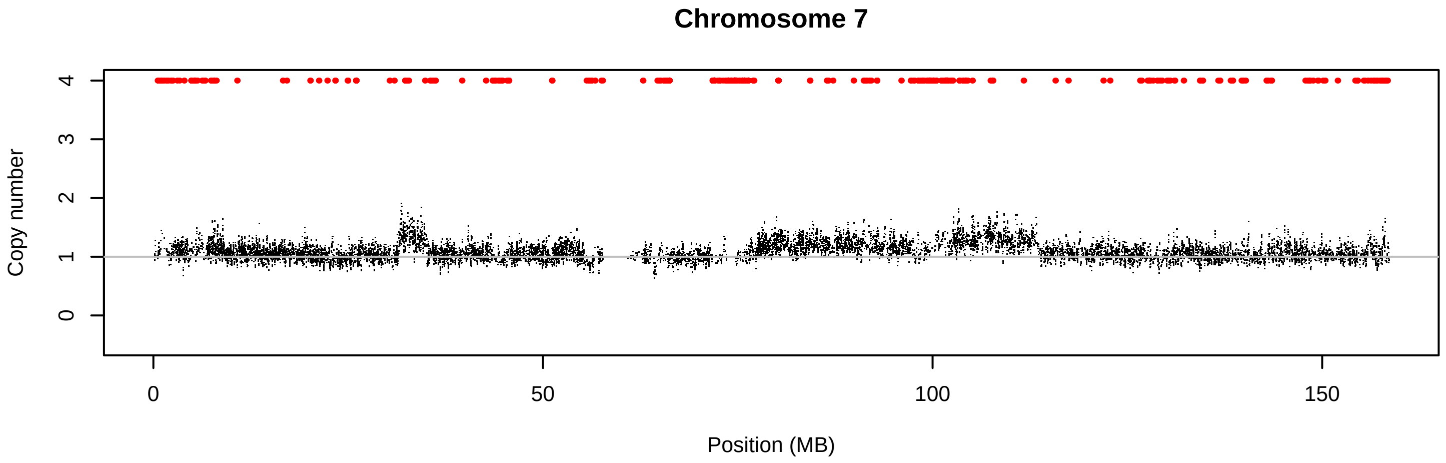
Chromosome 7
Copy number
0
1
2
3
4
0
50
100
150
Position (MB)

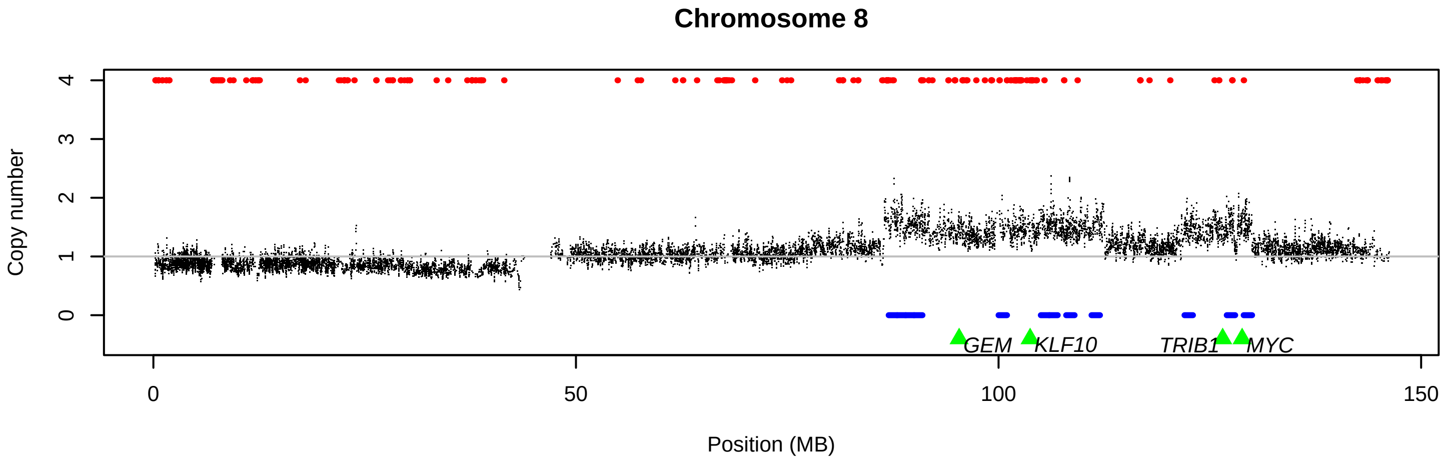
Chromosome 8
Copy number
0
1
2
3
4
GEM
KLF10
TRIB1
MYC
0
50
100
150
Position (MB)

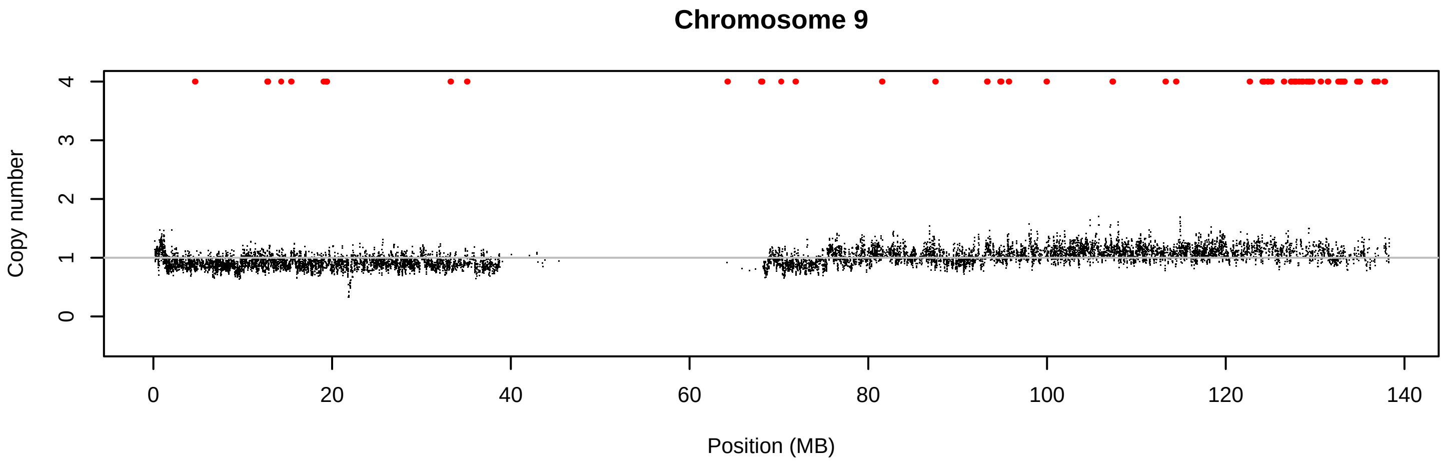
Chromosome 9
Copy number
0
1
2
3
4
0
20
40
60
80
100
120
140
Position (MB)

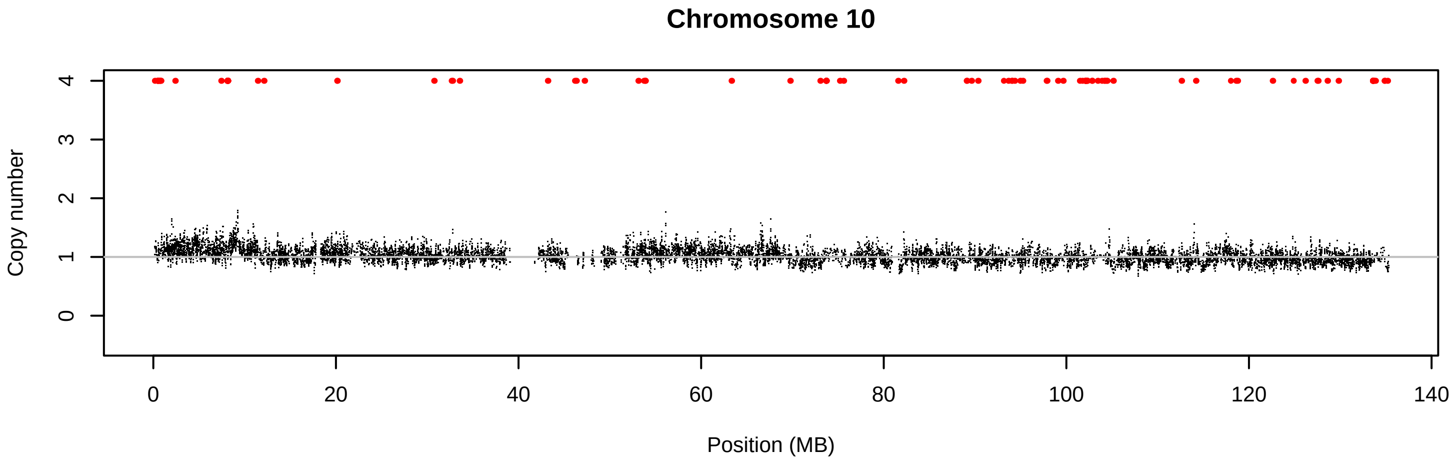
Chromosome 10
Copy number
0
1
2
3
4
0
20
40
60
80
100
120
140
Position (MB)

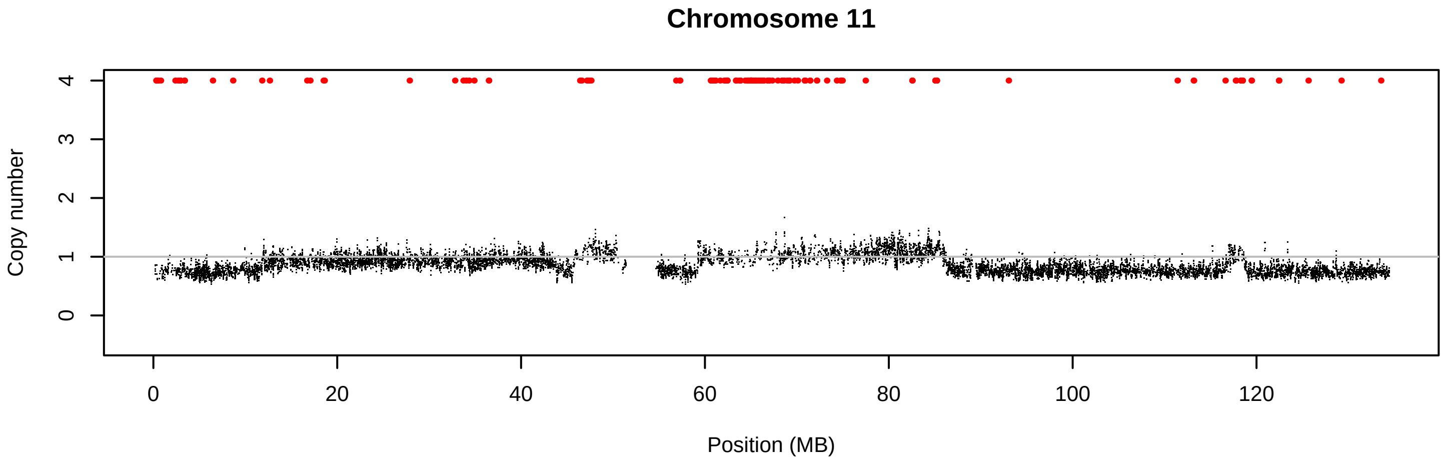
Chromosome 11
Copy number
0
1
2
3
4
0
20
40
60
80
100
120
Position (MB)

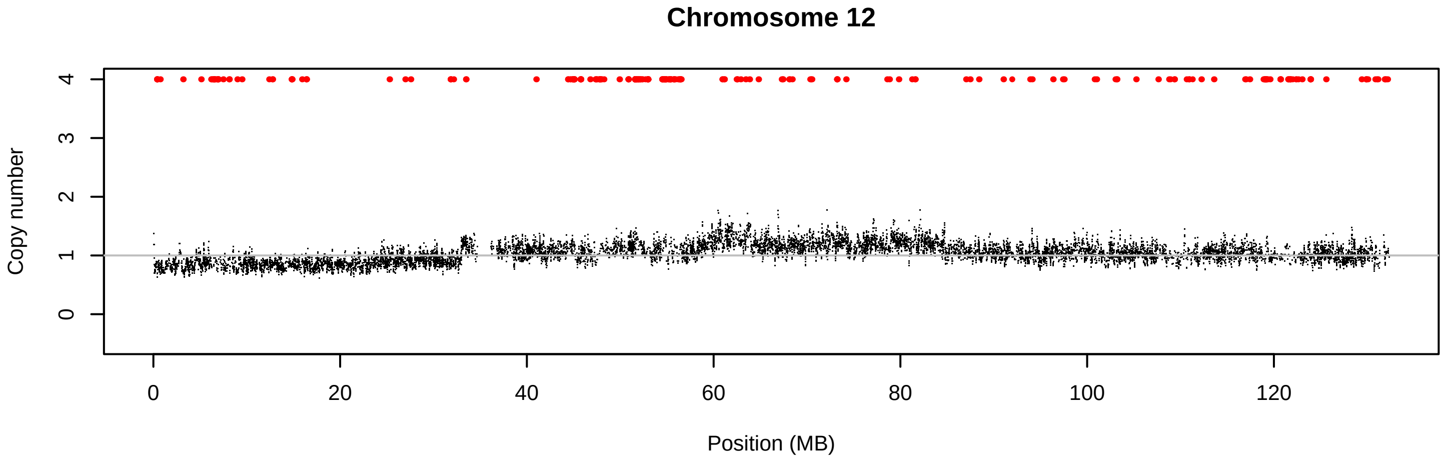
Chromosome 12
Copy number
0
1
2
3
4
0
20
40
60
80
100
120
Position (MB)

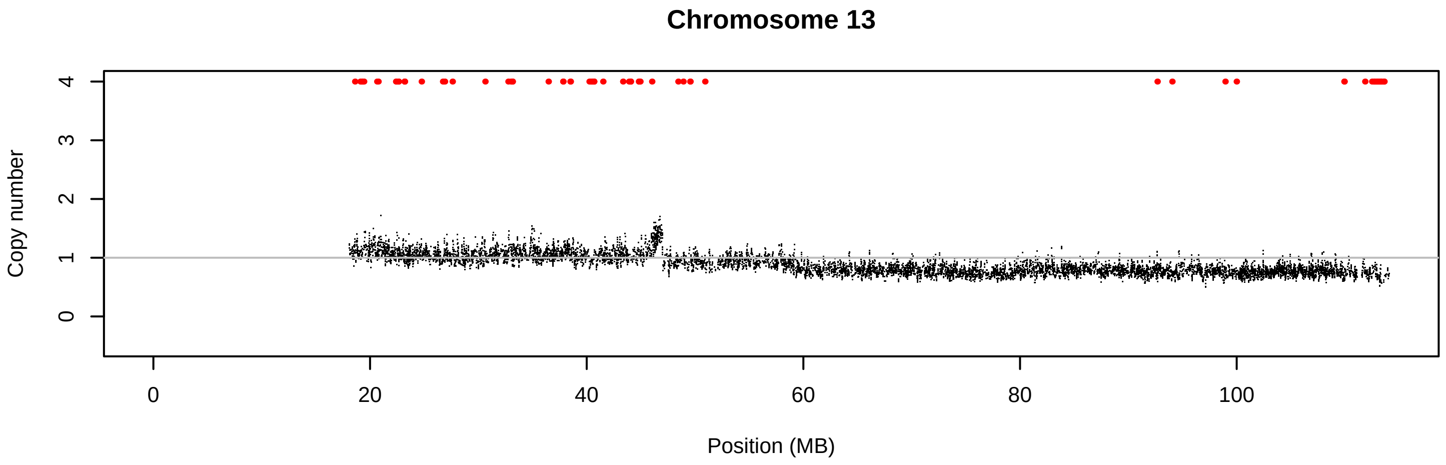
Chromosome 13
Copy number
0
1
2
3
4
0
20
40
60
80
100
Position (MB)

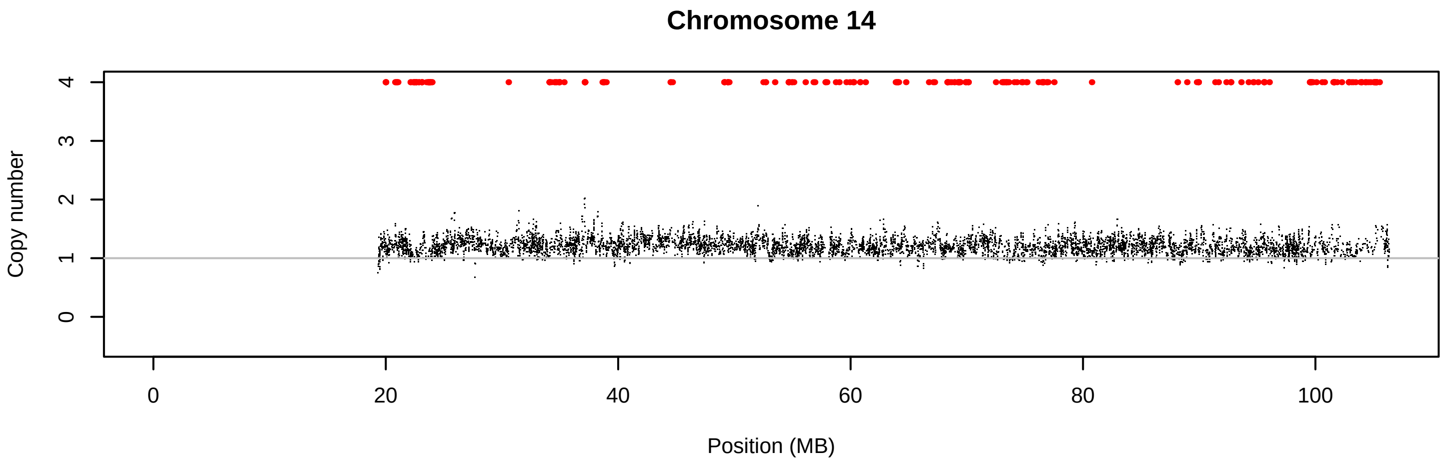
Chromosome 14
Copy number
0
1
2
3
4
0
20
40
60
80
100
Position (MB)

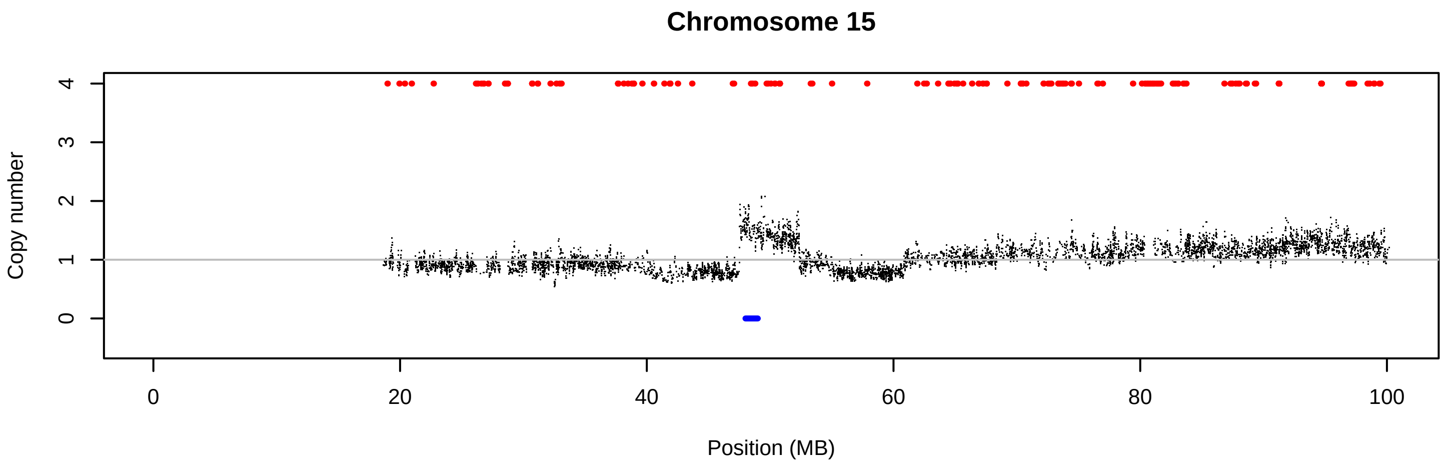
Chromosome 15
Copy number
0
1
2
3
4
0
20
40
60
80
100
Position (MB)

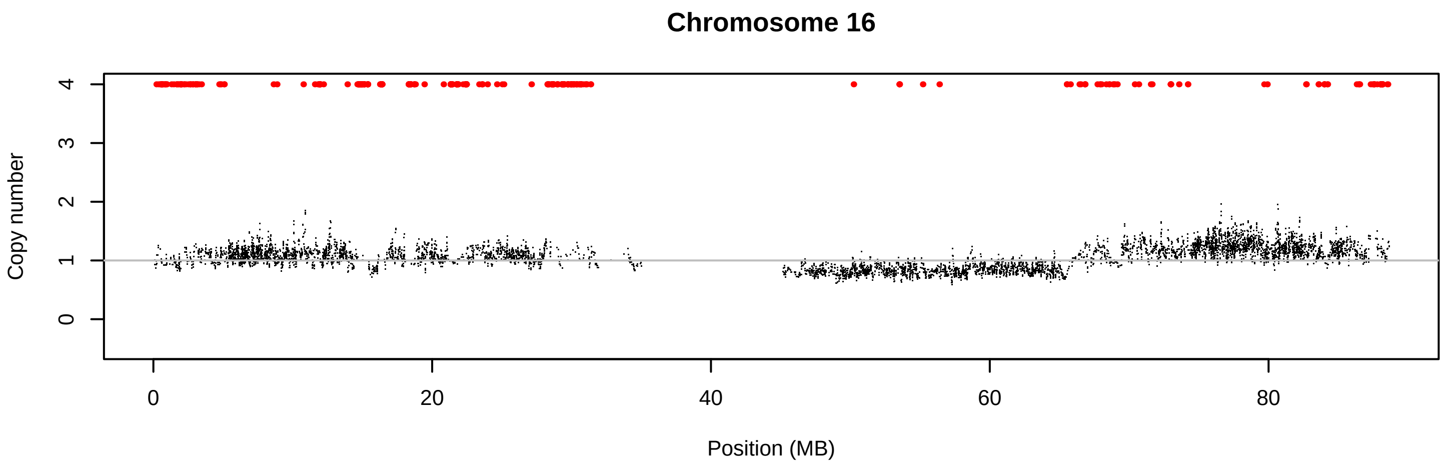
Chromosome 16
Copy number
0
1
2
3
4
0
20
40
60
80
Position (MB)

## Chromosome 17

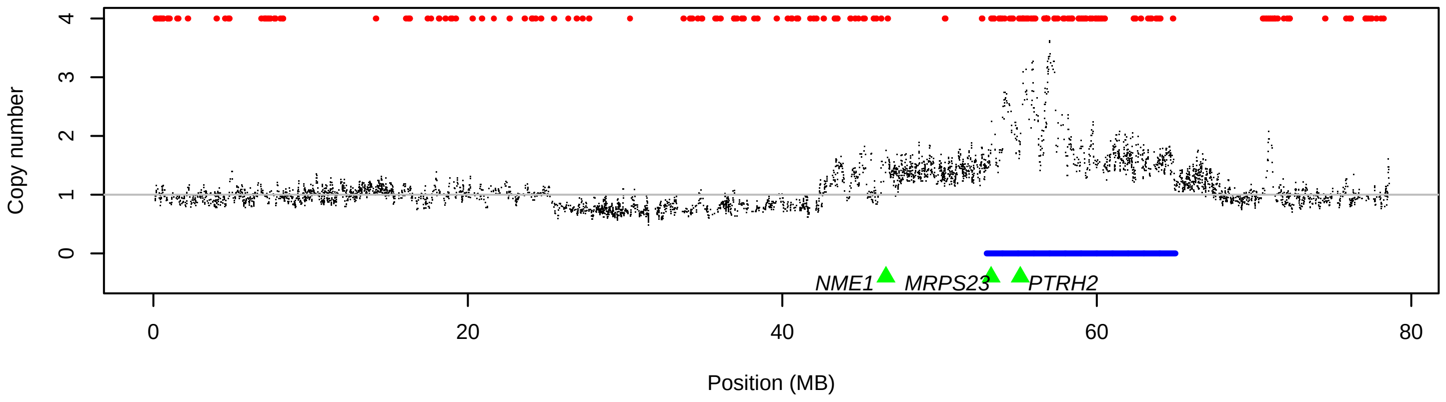


## Chromosome 18

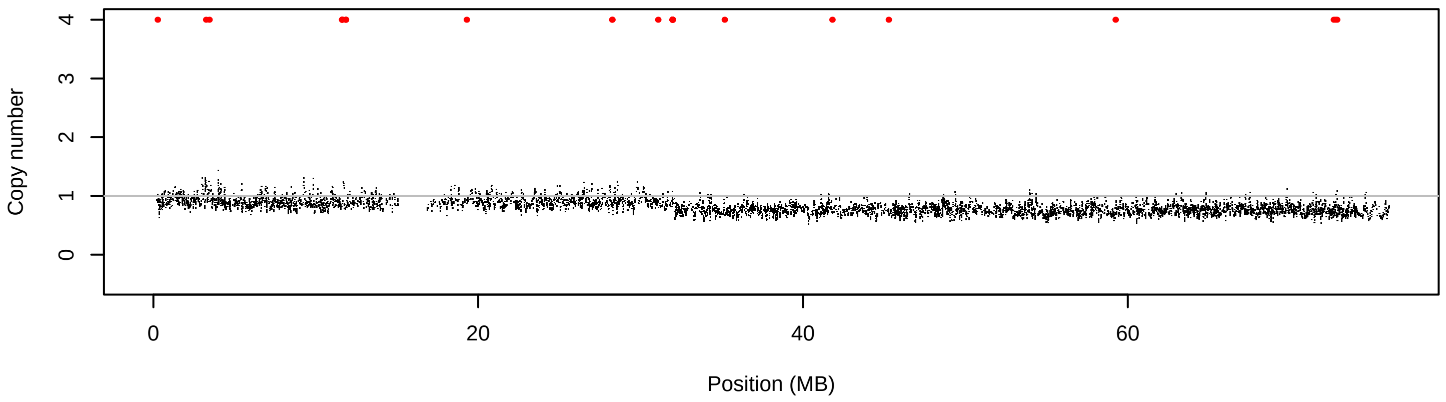


## Chromosome 19

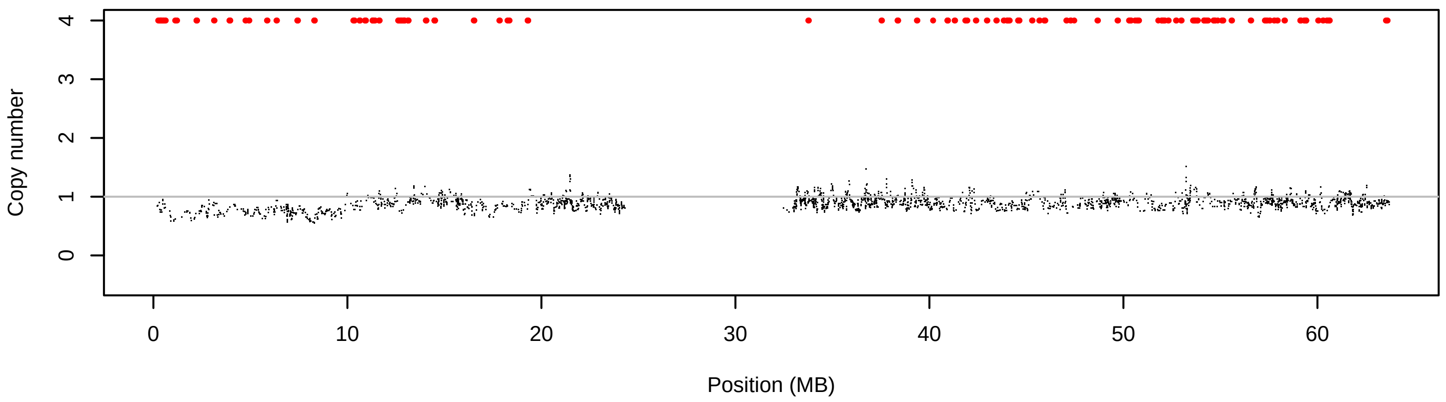


## Chromosome 20

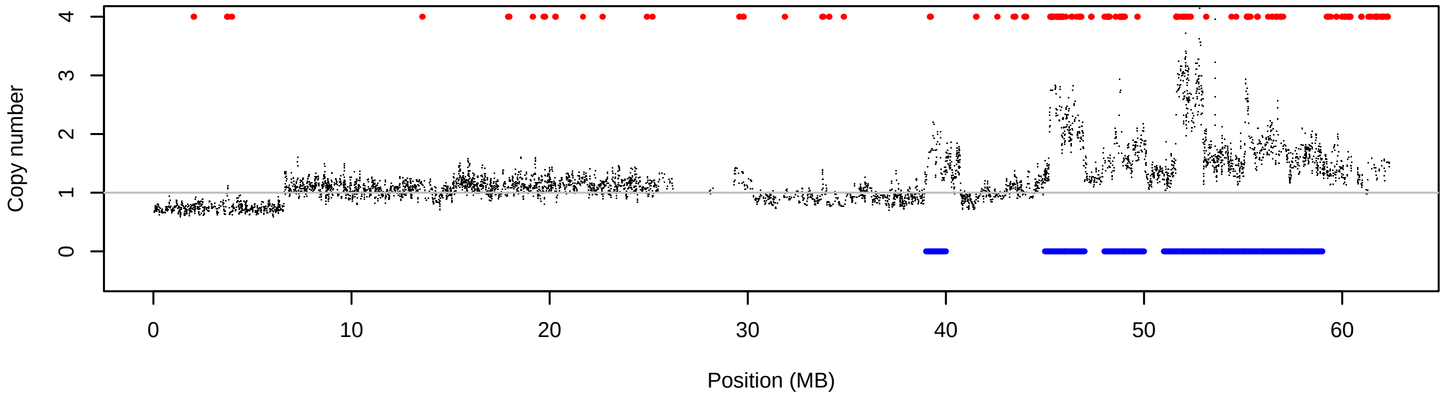

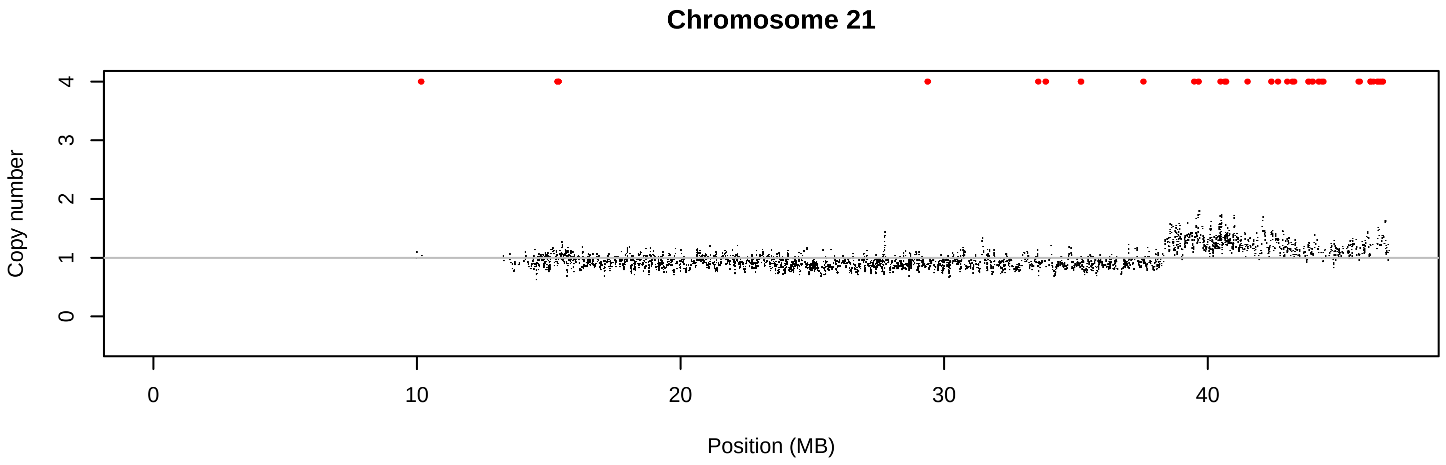

Chromosome 21
Copy number
0
1
2
3
4
0
10
20
30
40
Position (MB)


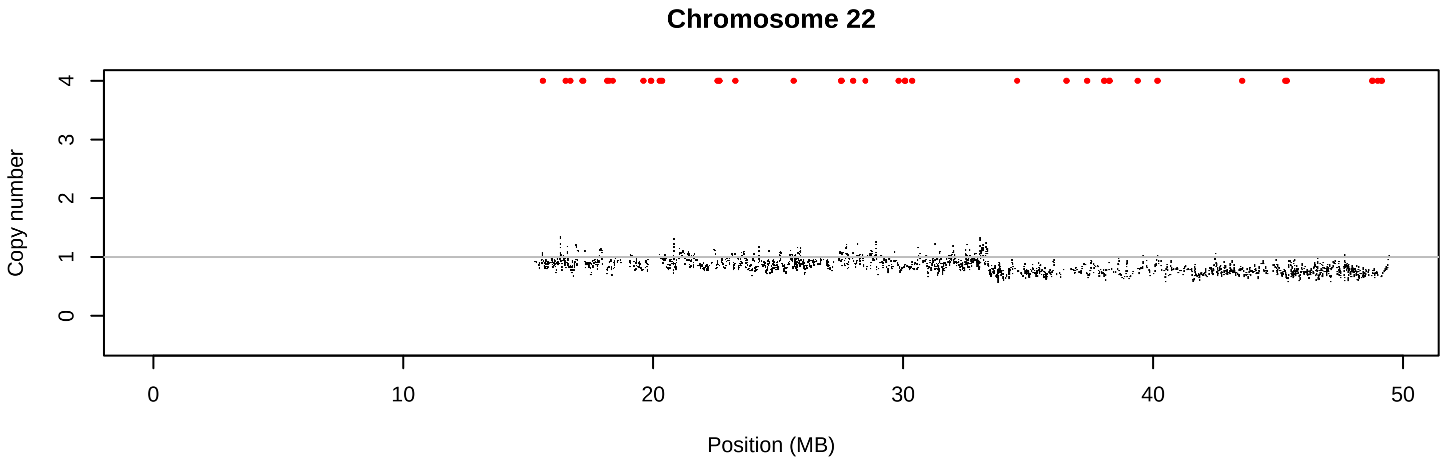

Chromosome 22
Copy number
0
1
2
3
4
0
10
20
30
40
50
Position (MB)
